# A nanodispersion-in-nanograins strategy for ultra-strong, ductile and stable metal nanocomposites

**DOI:** 10.1038/s41467-022-33261-5

**Published:** 2022-09-23

**Authors:** Zan Li, Yin Zhang, Zhibo Zhang, Yi-Tao Cui, Qiang Guo, Pan Liu, Shenbao Jin, Gang Sha, Kunqing Ding, Zhiqiang Li, Tongxiang Fan, Herbert M. Urbassek, Qian Yu, Ting Zhu, Di Zhang, Y. Morris Wang

**Affiliations:** 1 State Key Laboratory of Metal Matrix Composites, Shanghai Jiao Tong University, Shanghai, 200240 China; 2 Woodruff School of Mechanical Engineering, Georgia Institute of Technology, Atlanta, Georgia 30332 USA; 3 Institute of New Materials and Processing, Guangdong Academy of Sciences, Guangzhou, 510000 China; 4 Physics Department and Research Center OPTIMAS, University Kaiserslautern, Erwin-Schrödinger-Straße, D-67663 Kaiserslautern, Germany; 5 Synchrotron Radiation Laboratory, Laser and Synchrotron Research Centre (LASOR), The Institute for Solid State Physics, The University of Tokyo, Hyogo, 679-5165 Japan; 6 School of Materials Science and Engineering, Gleiter Institute of Nanoscience, Nanjing University of Science and Technology, Nanjing, 210094 China; 7 Center of Electron Microscopy and State Key Laboratory of Silicon Materials, School of Materials Science and Engineering, Zhejiang University, Hangzhou, 310027 China; 8 Department of Materials Science and Engineering, University of California, Los Angeles, CA 900095 USA

**Keywords:** Mechanical properties, Composites

## Abstract

Nanograined metals have the merit of high strength, but usually suffer from low work hardening capacity and poor thermal stability, causing premature failure and limiting their practical utilities. Here we report a “nanodispersion-in-nanograins” strategy to simultaneously strengthen and stabilize nanocrystalline metals such as copper and nickel. Our strategy relies on a uniform dispersion of extremely fine sized carbon nanoparticles (2.6 ± 1.2 nm) inside nanograins. The intragranular dispersion of nanoparticles not only elevates the strength of already-strong nanograins by 35%, but also activates multiple hardening mechanisms via dislocation-nanoparticle interactions, leading to improved work hardening and large tensile ductility. In addition, these finely dispersed nanoparticles result in substantially enhanced thermal stability and electrical conductivity in metal nanocomposites. Our results demonstrate the concurrent improvement of several mutually exclusive properties in metals including strength-ductility, strength-thermal stability, and strength-electrical conductivity, and thus represent a promising route to engineering high-performance nanostructured materials.

## Abstract (web summary)

High-strength nanocrystalline materials come at the expense of tensile ductility, thermal stability, and electrical conductivity. Here the authors report a nanodispersion-in-nanograins strategy where ultra-nano-carbon was used to concurrently achieve above four mutually exclusive properties.

## Introduction

High-strength materials are often compromised by the lack of work hardening, making them susceptible to cracking and catastrophic failure^1^. Nanocrystalline metals (with an average grain size less than 100 nm), for example, have been extensively investigated to achieve high strength. However, they exhibit low work hardening ability, leading to limited tensile ductility, together with low thermal stability and poor electrical conductivity. The alloying approach by one^2^ or multiple^3–5^ elements can, in principle, address some of the aforementioned limitations, but often comes with the high cost and further reduction of electrical conductivity. It also faces challenging issues of sustainability^6^ and sometimes intergranular embrittlement due to elemental segregation at grain boundaries (GBs). An alternative approach to strengthen fine-grained materials is to utilize nanosized reinforcements^7–12^. The resultant nanocomposites can exhibit enhanced strength, thermal stability, and electrical conductivity^10,12,13^. However, these reinforcements tend to agglomerate or distribute along GBs, causing local stress concentration, interface debonding, and premature failure^9,14,15^. As such, it remains challenging to achieve simultaneous improvements in strength, ductility, and thermal stability in nanograined metals – a class of high-strength materials with broad potential applications.

Here we report a “nanodispersion-in-nanograins” strategy to achieve enhanced mechanical properties and thermal stability along with enhanced electrical conductivity in nanocrystalline metals. Different from the commonly adopted approaches such as hierarchical microstructural design and GB engineering^16^, we achieve a uniform intragranular dispersion of a high density of carbon nanoparticles. Finely dispersed intragranular nanoparticles not only amplify the strengthening effect of nanograins but also activate multiple hardening mechanisms via dislocation-nanoparticle interactions, thereby rendering unique combinations of high strength, work hardening, and tensile ductility in nanocrystalline metals. Carbon is abundant in nature, low-cost, light, and has excellent mechanical and functional properties. We demonstrate the nanodispersion-in-nanograins strategy by introducing carbon nanoparticles into nanocrystalline copper (nc-Cu) and nickel (nc-Ni). Our nc-Cu composite has a high density (5.6 × 10^23^ m^−3^) of nanocarbon (2.6 ± 1.2 nm in particle size) inside Cu nanograins (63 ± 16 nm in grain size). Such intragranular nanodispersion enables a two-stage hardening mechanism in nanograined metals, thereby enhancing both strength and ductility. The dense nanodispersion further provides strong resistance to grain coarsening. As a result, the nc-Cu composites (e.g., 0.8 vol.% C) achieve an excellent combination of high tensile strength (1252 ± 22 MPa), uniform elongation (13.3 ± 0.9%), and high thermal stability (stable up to 0.72*T*_m_ for 1 h, where *T*_m_ is the melting temperature of Cu), together with an improved electrical conductivity. Our strategy thus represents an effective means of breaking trade-offs between several mutually exclusive properties in nanograined metals^17^. We also use the same approach to produce nc-Ni composites with outstanding properties, underscoring the general applicability of our nanodispersion-in-nanograins strategy for engineering high-performance nanostructured metals.

## Results

**Fig. 1:**
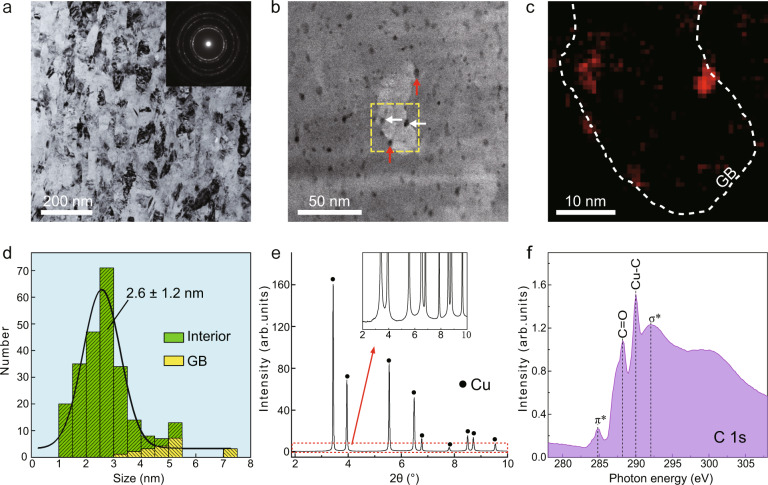
Microstructure of nc-Cu composites dispersed with unc particles. **a** A bright-field (BF) TEM image showing nanocrystalline grains in an as-synthesized composite, as confirmed by nearly uniform diffraction rings in the inset. An aperture size of 1 μm was used. **b** An HAADF-STEM image of nc-Cu with a C concentration of 0.8 vol.%, where a high density of unc particles is observed. Some unc particles within grains and along grain boundaries (GBs) are indicated by white and red arrows, respectively. BF- and HAADF-STEM images are used together to identify GBs. **c** EELS C imaging map for the boxed area in **b** where the GB is delineated by the white dashed line. **d** A histogram showing the size distribution of unc particles and their locations either in grain interiors or at GBs, as measured by STEM; 92% of 252 unc particles are found inside grains. **e** Synchrotron X-ray diffraction (SXRD) pattern of the composite. Inset is the magnified spectrum of the boxed region, showing the absence of additional peaks except for those of Cu. No carbide forms at Cu-C interfaces. **f** Near-edge x-ray absorption fine-structure (NEXAFS) spectrum at the C-K edge of nc-Cu composite (0.8 vol.% C). A distinctive peak (~290 eV) arising from Cu-C covalent bonding is observed.

The intragranular dispersion of nanoparticles in nanograins is difficult, as nanoparticles tend to agglomerate or trap at GBs. To overcome these challenges, we developed a chemical bond-assisted dispersion strategy to enable the fragmentation and embedding of nanoparticles. High-energy ball milling was applied to metal nanoflakes with defective reduced graphene oxide (RGO) uniformly distributed on nanoflake surfaces (Methods). First principles calculations indicate that chemical metal-carbon bonding forms at the graphene-metal interface where dangling carbon bonds exist (Supplementary Fig. 1), thus promoting strong interfacial bonding. Such metal-carbon bonding can suppress interfacial sliding of RGO on metal surfaces upon ball milling, leading to the rapid fragmentation and effective embedding of carbon nanoparticles (Methods). Microstructural characterization by transmission electron microscopy (TEM) in Fig. 1a–c indicates that as-synthesized nc-Cu is embedded with finely dispersed carbon nanoparticles. The average grain size of nc-Cu is 63 ± 16 nm, Fig. 1a. A high density of uniformly distributed carbon nanoparticles, up to a carbon concentration of 0.8 vol.%, is shown by a high-angle annular dark-field scanning transmission electron microscopy (HAADF-STEM) image in Fig. 1b and electron energy loss spectroscopy (EELS) carbon imaging map in Fig. 1c. The statistical histogram in Fig. 1d indicates that the carbon nanoparticles have an average diameter of 2.6 ± 1.2 nm and a number fraction of ~92% inside grains as opposed to at GBs. Because of their extremely small sizes (<5 nm), the carbon nanoparticles are referred to as ultra-nano-carbon (unc)^18^. We demonstrated the efficacy of this dispersion strategy by experimenting less-defective nanocarbon (including 1D nanotubes and 2D nanosheets, see Methods) in nanograins; correspondingly, most nanocarbon was found at GBs, likely due to the weak bonding between metal and less-defective nanocarbon that results in easy sliding of nanocarbon on metal surfaces (Supplementary Fig. 2).

The bonding nature of unc with the metal matrix was investigated using synchrotron X-ray diffractometry (SXRD) and near-edge X-ray absorption fine-structure (NEXAFS) spectroscopy. Raman analysis shows that unc particles have an average crystalline domain size of ~1.1 nm (see Methods), suggesting their highly defective nature. SXRD results in Fig. 1e indicate a single face-centred cubic phase of the Cu matrix, where no carbon peaks and compounds were detected. We applied NEXAFS (Fig. 1f) to further analyse the Cu-C bonding. Apart from the spectral features of C 1s - π^*^/σ^*^ transitions of core electrons into unoccupied states, two main additional peaks are identified. One is an adsorption peak resulting from C-O interactions (288 eV) inherited from residual functional groups in RGO, and the other is an adsorption peak near 290 eV attributed to Cu-C chemisorption. The observation of strong Cu-C bonding should not be attributed to Cu-C compounds, as Cu is the only crystalline phase identified in the SXRD pattern of the composite (Fig. 1e). The origin of this Cu-C bonding can be inferred from first principles simulations (Supplementary Fig. 1), in which chemical Cu-C bonding is found to be localized at defective sites without forming long-range ordering, in accordance with the synchrotron results.

**Fig. 2:**
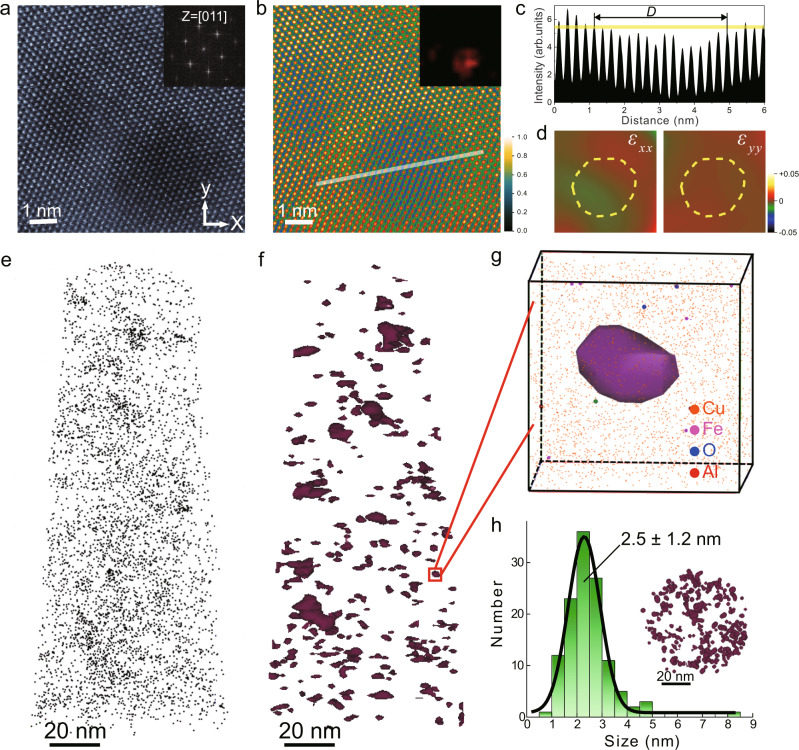
High-resolution TEM and APT characterization of nc-Cu composite (0.8 vol.% C). **a** A high-resolution HAADF-STEM image of the local atomic structure near unc particles taken along the [011] zone axis, as confirmed by the fast Fourier transformation (FFT) pattern in the inset. **b** FFT-filtered image of **a**. Variation of colour from black to yellow indicates intensities from low to high. Inset is the C imaging map for this region, showing that the low-intensity regions correspond to unc (due to the sensitivity of STEM image to atomic number Z). Inset shows two unc particles determined from their FFT image (see **c** for clarity). **c** Intensity profile along the line in **b**, where the decrease of atom intensity in the unc-contained region is observed. The yellow line, which represents 90% of the averaged atom intensity of the Cu matrix, is taken as the cutoff intensity to estimate the diameter (4.1 nm) of the unc particle. **d** Strain maps of *ε*_*xx*_ and *ε*_*yy*_ in the unc-contained region in **a**. The reference zero strain region is chosen from the dislocation free region (the upper-left corner of **a**). No obvious strain concentration is observed near the unc particle (marked by a dashed-line circle). **e**–**f** Side-view APT images of C background and unc particles reconstructed with the threshold iso-composition surface below and above 0.25 at.% C, respectively. **g** Enlarged region in **f** showing a representative unc particle, along with atomic distributions of Cu (10% of Cu atoms are shown to reduce the background intensity) and other detected impurities, including Al, Fe and O. **h** A statistical histogram showing the size distribution of unc particles (124 counts). The average particle size is 2.5 ± 1.2 nm. Inset shows a top-view APT image of unc particles, indicating their nearly spherical shapes.

High-resolution TEM and atom probe tomography (APT) were used to characterize unc particles (Fig. 2). The HAADF-STEM image in Fig. 2a indicates that the Cu-C interface is clean, and no significant lattice distortion is present surrounding unc particles. The individual particle size can be determined under HAADF-STEM by combining the fast Fourier transformation (FFT) filtered image and the intensity variation of lattice fringes (Fig. 2b and c). This is due to the sensitivity of STEM image to the atomic number (Z). Geometrical phase analysis (GPA) strain mapping further indicates little distortion at the Cu-C interfaces, as the atomic strains around unc particles exhibit nearly uniform distributions (Fig. 2d). APT measurements (Fig. 2e–h) show that the number density of unc particles reaches a high mean value of ~5.6 × 10^23^ m^−3^. Most unc particles are nearly spherical in shape and show random distributions, as revealed by the side-view (Fig. 2f) and top-view APT images (inset of Fig. 2h) (Supplementary Movie 1). The average size of unc particles from APT probe is 2.5 ± 1.2 nm (Fig. 2h), in agreement with TEM measurements. Some trace amounts of impurities (i.e., Fe, Al, O) are detectable but no large clusters are found (Fig. 2g); limited C atoms are dissolved in the Cu matrix (0.01 at.%, Fig. 2e), due to the low solubility of C in Cu.

**Fig. 3:**
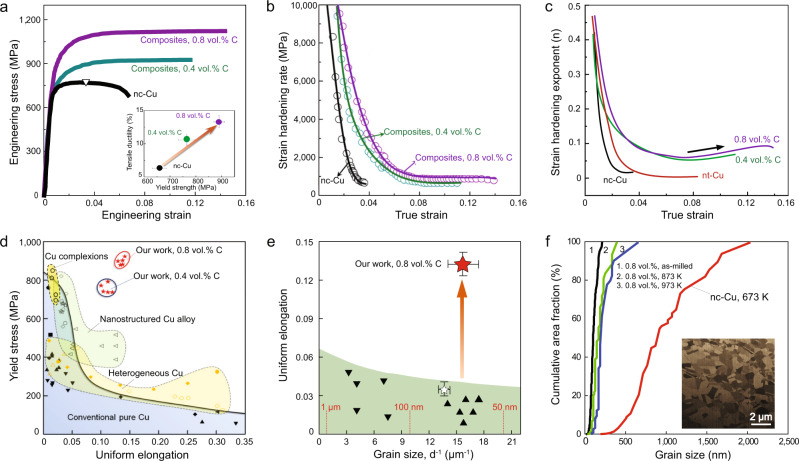
Tensile properties and thermal stability of nc-Cu composites. **a** Representative tensile engineering stress-strain curves for pure nc-Cu and nc-Cu composites (0.4 vol.% and 0.8 vol.% C, respectively). All tensile tests were performed at a strain rate of 5 × 10^−4^ s^−1^ and room temperature. The strain at which necking occurs is marked with an open triangle for pure nc-Cu. The inset shows the simultaneous improvement of yield strength and uniform elongation of nc-Cu composites over pure nc-Cu. **b** Experimentally measured strain hardening rate *dσ/dε* (with *σ* and *ε* being the true stress and true strain, respectively) for nc-Cu composites and nc-Cu. **c** Strain hardening exponent (*n* = d(ln*σ*)*/*d(ln*ɛ*)) as a function of strain. The reference nt-Cu plot was calculated from the literature^26^. **d** Yield strength versus uniform elongation of nc-Cu composites as compared with those of other Cu-based materials, including heterogeneous Cu, nanostructured Cu alloys and complexion-engineered Cu. Superior properties are observed for nc-Cu composites. Sources of the references are cited in the Methods. **e** Uniform elongation versus grain size of nc-Cu composites as compared with those of pure nc-Cu. **f** Thermal stability of nc-Cu composites: cumulative area fraction of grain size of the nc-Cu composite (0.8 vol.% C) annealed at various temperatures (873 K and 973 K) for 1 h, in comparison with that of the reference nc-Cu annealed at 673 K for 1 h. Inset shows pronounced grain growth in nc-Cu, as indicated by the ion-channelling cross-sectional image. In contrast, no grain growth was observed in nc-Cu composites after annealing at 973 K for 1 h.

We conducted in situ scanning electron microscope (SEM) micro-tensile tests to measure the mechanical properties of nc-Cu composites (Supplementary Fig. 3, Supplementary Movie 2, and Movie 3). As-prepared tensile samples have a sufficiently large number of Cu nanograins (~200) through the cross section (1.3 μm × 1.3 μm), thus minimizing the sample size effect on measured mechanical properties^19,20^. Two approaches, namely elastic strain calibration and dynamic image tracking, were employed to ensure the validity of strain measurement during tensile testing (Methods). Five samples of each type were tested to confirm the reproducibility of measurements (Supplementary Fig. 4a–c). Figure 3a shows the representative tensile stress-strain curves of pure nc-Cu (with the average grain size of 73 ± 16 nm), and two nc-Cu composites reinforced by 0.4 vol.% and 0.8 vol.% of unc particles, respectively. The reference nc-Cu has a yield strength (*σ*_y_) of 660 ± 28 MPa, ultimate tensile strength (*σ*_u_) of 776 ± 21 MPa, and elongation to failure (*ε*_f_) of 6.3% ± 0.4%, which are consistent with those reported for bulk nc-Cu samples with similar grain sizes that were processed by in situ consolidation as well^21^. For our nc-Cu composites, *σ*_y_ increases to 765 ± 32 MPa and 890 ± 21 MPa for the carbon volume fraction of 0.4% and 0.8%, respectively. Beyond plastic yielding, nc-Cu composites are further work-hardened until failure, giving the large *σ*_u_ of 995 ± 11 MPa and 1252 ± 22 MPa for 0.4 vol.% and 0.8 vol.% of unc, respectively, with the corresponding large uniform elongation (*ε*_u_) of 10.6% ± 0.8% and 13.3% ± 0.9%. Note that the *ε*_u_ of our nc-Cu composite is superior to that recently reported in a nanocrystalline alloy achieved by composition undulation^22^. The *σ*_u_ approaches the strength limit of nanocrystalline/nanotwinned Cu^23^ when the grain size/twin spacing is extremely small (~10 nm)^24,25^.

The large tensile ductility in nanocrystalline composites is attributed to their high work hardening rates, as shown in Fig. 3b. Compared to pure nc-Cu, nc-Cu composites exhibit much stronger hardening rates at small strains and retain higher hardening rates at large strains. The instantaneous work-hardening exponent^5^ (*n*) shows an ‘up-turn’ behaviour in nc-Cu composites at large strains (~7%) (Fig. 3c), which is absent in pure nc-Cu and thus implies the operation of additional hardening mechanisms arising from unc. The strong work hardening responses in nc-Cu composites push the limit of the strength-ductility combination beyond other strengthening strategies for Cu such as grain refinement, alloying, and incorporation of special structures/interfaces (Fig. 3d). By using the nanodispersion strategy, our nc-Cu composite stands outside the conventional regime of ductility versus grain size (Fig. 3e). It also demonstrates superior tensile properties over Cu nanocomposites made via other methods, where a high degree of intragranular dispersion of reinforcements is not attainable (Supplementary Fig. 5). Note that nanotwinned Cu (nt-Cu) possesses an excellent combination of high strength and ductility^26^. However, the *σ*_u_ and *ε*_u_ of our nc-Cu composite (0.8 vol.% C) are 17% and 62% higher, respectively. It is unlikely that the high strengths of our nc-composites originate from impurities introduced during ball milling, as only a very small trace amount of impurities is present in our samples (Fig. 2g and Supplementary Table 1). Ga ions may be introduced during tensile specimen preparation, but their impact on tensile strengths is negligible (Supplementary Fig. 4d, e). Micro-tensile samples with larger dimensions were measured with nearly identical tensile properties (Supplementary Fig. 4f), confirming a negligible sample size effect. Macroscopic tensile tests were also conducted for bulk pure nc-Cu and composites by densifying the powders (Methods). These macro-tensile tests reveal similarly high stress and ductility to the micro-tensile tests (Supplementary Fig. 6).

**Fig. 4:**
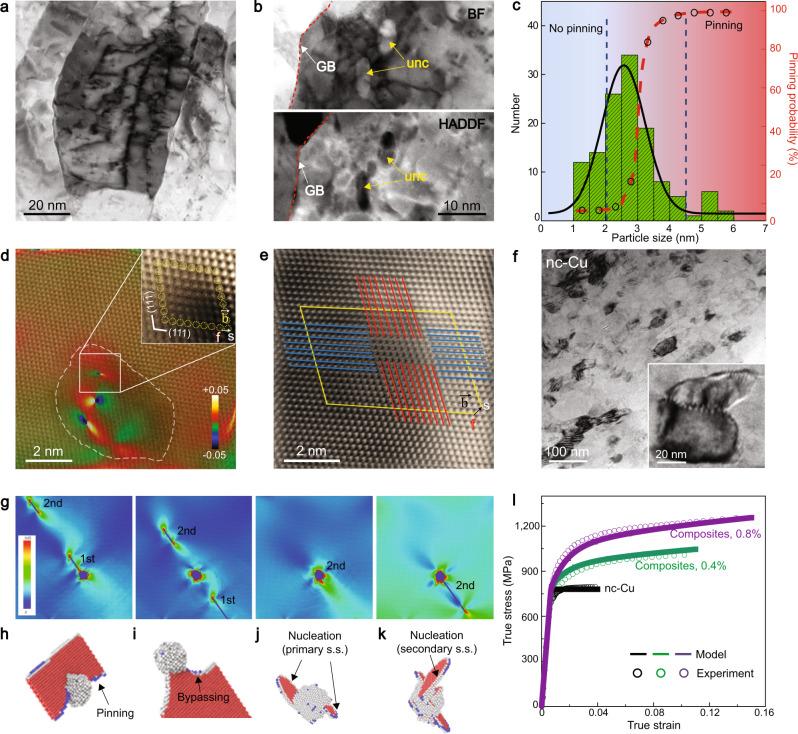
Strengthening and hardening mechanisms in nc-Cu composites. **a** BF**-**STEM image of an nc-Cu composite (0.8 vol.% C) after tensile fracture. A high density of dislocations was stored inside nanograins. **b** BF- and HAADF-STEM images of the same region. **c** Size distribution histogram of unc particles and corresponding dislocation pinning probability. 127 particles were examined. Unc particles with larger diameters are found to have a higher probability of dislocation pinning. **d** A high-resolution HAADF-STEM image shows atomic structure near a unc particle, superimposed with the dilatation strain (*ε*_*xx*_) map. The zero-strain reference was chosen in the lower-left corner of this image. Three dislocation cores can be identified at the C nanoparticle region (indicated by the yellow dashed circle). The Burgers circuit analysis (inset) of one dislocation indicates the Burgers vector of 1/2[011]. **e** High-resolution HAADF-STEM image of the deformed microstructure away from unc particles. Two full dislocations on different {111} planes form an immobile Lomer lock, as revealed by the Burgers circuit analysis. **f** BF**-**STEM image of the tensile fractured pure nc-Cu. Few dislocations were stored. Inset is a representative grain after deformation. **g** Snapshots of MD simulations for successive dislocation bypassing across a periodic array of unc particles aligned in the out-of-plane direction. Each dislocation dissociates into a pair of leading and trailing partials with a stacking fault in between. Atomic von Mises strain maps in **g** reveal the local strain fields around dislocations and unc particles as well as long-range elastic interactions between dislocations. **h**–**k** Magnified MD snapshots showing the 3D dislocation structure near a unc particle during the pinning of the leading partial (**h**), and the depinning of the trailing partial (**i**). **j** Nucleation of a dislocation loop consisting of two half loops from the unc particle on the primary slip system with the largest resolved shear stress. The two half loops form on parallel {111} slip planes. **k** Nucleation of a dislocation loop on the secondary slip system with a lower resolved shear stress. Atoms in **h**–**k** are coloured through common neighbour analysis. **l** Stress-strain responses obtained from the two-stage hardening model, in comparison with experimental results.

We investigated the strengthening and hardening mechanisms of nc-Cu composites by combining the TEM characterization and atomistic modelling of dislocation-unc interactions. Figure 4a presents a bright field (BF)-STEM image showing curved and tangled dislocations in a deformed nc-Cu composite (0.8 vol.% C). A higher magnification STEM image in Fig. 4b shows that dislocations are accumulated near the Cu-C interface, suggesting the strong pinning effect of unc particles. From statistical analysis, we estimated the critical particle size of ~3 nm above which strong dislocation pinning occurs (Fig. 4c); such critical size is smaller than those for precipitates in conventional peak-aged Cu-based alloys (>5 nm)^27,28^. This result suggests that the intragranular unc renders a higher pinning resistance than crystalline precipitates. To understand this effect, our molecular dynamics (MD) studies show that the critical stress for dislocation bypassing of unc particles increases with interfacial Cu-C strength (Supplementary Fig. 7). Thus, dislocation pinning by unc is enhanced by strong Cu-C bonding, elevating the strength of already-strong nanograins. Atomic resolution HAADF-STEM imaging further reveals dislocation storage near unc. In Fig. 4d, three dislocations with different local strain fields around their core structures are identified, suggesting the operation of multiple slip systems inside the nanograins; they are full dislocations as shown by a Burgers circuit analysis in the inset of Fig. 4d. The obstructed dislocations at unc particles are observed from HAADF-STEM images taken along different zone axes (e.g., [001], Supplementary Fig. 8). Moreover, Fig. 4e shows a dislocation lock away from unc, which results from interlocking of dislocations on two different slip systems. Therefore, the nc-Cu composites enable dislocation storage inside the confined volume of nanograins, elevating the strain hardening ability. In contrast, during straining of pure nc-Cu, a dislocation nucleated from a GB usually encounters a limited number of intragranular obstacles as it travels across a nanograin and becomes absorbed into an opposite GB, which leads to insignificant dislocation storage inside nanograins, as indicated in Fig. 4f.

We further performed MD simulations to investigate the dislocation pinning and accumulation mechanisms associated with unc particles^29–32^. MD results in Fig. 4g show the dynamic processes of two $1/2\langle 110\rangle \{111\}$ full dislocations (marked as 1^st^ and 2^nd^) that sequentially bypass a periodic array of unc particles embedded in a Cu matrix. The spherical unc particles have a diameter *d* of 2.5 nm and a spacing *l* of 10.7 nm, giving an equivalent C concentration of about 0.8 vol.%. Atomic strain maps in Fig. 4g reveal the strong pinning effects of unc particles on the two dislocations. Specifically, the obstructed 1^st^ dislocation exerts strong back stress to the 2^nd^ dislocation, due to their long-range elastic interaction manifested as interconnected light-green strain contours. This back stress opposes the motion of the 2^nd^ dislocation, giving rise to a strong back-stress hardening effect arising from unc particles^33^ and thus contributing to the strong work hardening of nc-Cu composites. Figure 4h, i present the corresponding three-dimensional process where the 1^st^ dislocation bypasses a representative unc particle via the Orowan mechanism^34^. After bypassing, an interface dislocation loop is left around the unc particle, producing a shell of high strain contour (in red). This loop corresponds to the geometrically necessary dislocation (GND)^29^. Similar MD results were obtained for both amorphous and diamond-cubic unc particles, as well as for random distributions of unc particles (Supplementary Fig. 9). An increase of applied load raises the GND density, thereby increasing the local stress concentration around unc particles. To release these stresses, additional dislocations can nucleate on other primary slip planes (Fig. 4j) as well as on secondary slip planes (Fig. 4k). These newly nucleated dislocations tend to spread out between unc particles with increasing load and thus promote dislocation interactions on multiple slip systems^29,32^, further contributing to the work hardening of nc-Cu composites.

Based on the above experimental and MD results, we developed a two-stage work hardening model to quantitatively evaluate the primary effects of unc on the experimental stress-strain responses, in order to determine major sources of the extra work hardening of nc-Cu composites relative to nc-Cu (Methods). During stage I hardening at small strains, the nc-Cu composites exhibit markedly higher hardening rates (Fig. 3b), quickly raising yield strengths by significant amounts relative to nc-Cu. This is because the density of GNDs (*ρ*^*G*^) increases quickly with applied strain due to the highly dense distribution of unc. Strong bonding at Cu-C interfaces hinders the relaxation of GNDs around unc particles. As a result, the partially relaxed GNDs collectively exert a large long-range back stress to the strength-limiting process which could involve the activation of dislocation sources near GBs^35^. To account for this strong hardening effect, the total slip resistance on the primary slip plane *τ*_T_ is represented approximately by ${\tau }_{{{{{{\rm{T}}}}}}}={\tau }_{{{{{{\rm{y}}}}}}}+{\tau }_{{{{{{\rm{I}}}}}}}^{{{{{{\rm{UNC}}}}}}},$ where *τ*_y_ is the shear resistance of the strength-limiting process for pure nc-Cu; ${\tau }_{{{{{{\rm{I}}}}}}}^{{{{{{\rm{UNC}}}}}}}$ is the back stress from the GNDs on the primary slip planes arising from unc particles. ${\tau }_{{{{{{\rm{I}}}}}}}^{{{{{{\rm{UNC}}}}}}}$ rises quickly with increasing *ρ*^*G*^ in a strong nonlinear manner, and becomes saturated at the tensile strain of a few percent; the saturated value of ${\tau }_{{{{{{\rm{I}}}}}}}^{{{{{{\rm{UNC}}}}}}}$ due to stage I extra work hardening from unc can result in an extra tensile stress of 206 MPa for 0.8 vol.% unc. Afterwards, stage II hardening sets in, due to nucleation and spread of additional dislocations on multiple slip planes. To account for this additional hardening effect, the total slip resistance is represented as ${\tau }_{{{{{{\rm{T}}}}}}}={\tau }_{{{{{{\rm{y}}}}}}}+{\tau }_{{{{{{\rm{I}}}}}}}^{{{{{{\rm{UNC}}}}}}}+{\tau }_{{{{{{\rm{II}}}}}}}^{{{{{{\rm{UNC}}}}}}}$, where ${\tau }_{{{{{{\rm{I}}}}}}}^{{{{{{\rm{UNC}}}}}}}$ has become saturated; ${\tau }_{{{{{{\rm{II}}}}}}}^{{{{{{\rm{UNC}}}}}}}$ is the slip resistance from dislocation interactions and increases with a lower rate than stage I due to the spread of these nucleated dislocations around unc particles^29^. The saturated value of ${\tau }_{{{{{{\rm{II}}}}}}}^{{{{{{\rm{UNC}}}}}}}$ due to stage II extra work hardening from unc can raise the extra tensile stress to 315 MPa for 0.8 vol.% unc relative to nc-Cu. As shown in Fig. 4l, the numerical results from the two-stage hardening model agree closely with the experimental stress-strain responses of nc-Cu composites and nc-Cu. Hence, ${\tau }_{{{{{{\rm{I}}}}}}}^{{{{{{\rm{UNC}}}}}}}$ and ${\tau }_{{{{{{\rm{II}}}}}}}^{{{{{{\rm{UNC}}}}}}}$ reflect major sources of the two-stage hardening behaviours arising from unc in nc-Cu composites; they underscore the important role of the nanoscale structural heterogeneities^36^ such as unc particles in enabling the high work hardening and tensile ductility of nanocrystalline composites.

For nc-Cu composites, the ultimate tensile strain increases with the ultimate tensile strength, which rises with increased unc content (up to 0.8 vol.% C) (Fig. 3a). To understand this counterintuitive trend, we note that the unc-induced dislocation interactions offer an effective mechanism to accommodate deformation incompatibility between the unc particles and Cu matrix, thus preventing the premature fracture initiated from unc particles. Furthermore, the unc-enhanced dislocation activities inside nanograins can benefit the accommodation of deformation compatibility between neighbouring grains. For pure nanocrystalline metals, dislocations tend to nucleate from a GB and glide through the associated nanograin before being absorbed at an opposite GB. As a result, deformation incompatibility and local stress concentrations could quickly build up near GBs, causing GB fracture and tensile failure of pure nc-Cu^37^. In contrast, a high density of unc particles in nc-Cu composites can obstruct dislocation glide inside nanograins, which exert back stresses to resist the continued operation of GB dislocation sources. The unc-induced activation of multiple slip systems inside nanograins also promotes dislocation interaction, strain hardening and deformation uniformity. Hence, these intragranular dislocation activities can slow down dislocation nucleation and absorption at GBs, reducing deformation incompatibility at GBs and thus delaying intergranular fracture in nc-Cu composites. For nc-Cu composites reinforced with less-defective nanocarbon, the unc particles tend to segregate at GBs. This effect reduces dislocation-particle interactions inside nanograins, rendering limited property enhancement (Supplementary Fig. 10).

**Fig. 5:**
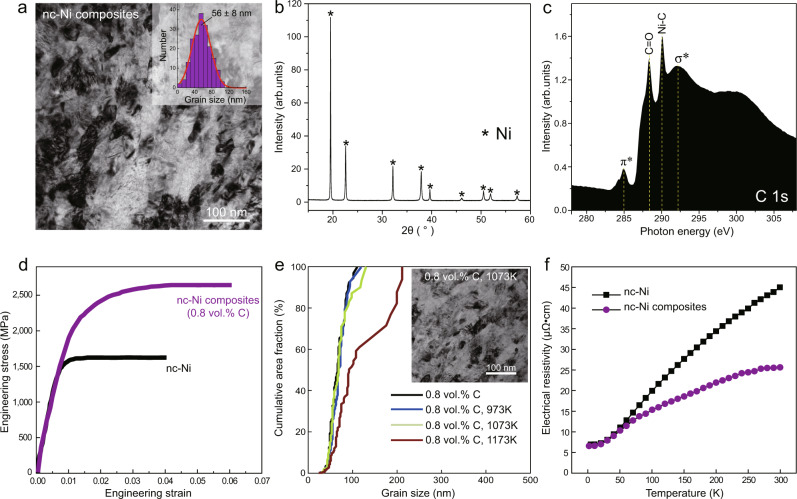
Microstructure, tensile properties, and thermal stability of pure nc-Ni and nc-Ni composite. **a** A bright-field TEM image of nc-Ni composite (0.8 vol.% C). Inset shows the Ni grain size distribution of the composite. The grain size of nc-Ni obtained with the identical fabrication process is 61 ± 10 nm**. b** SXRD pattern of the composite. Note that no Ni-C compounds are observed in SXRD experiments. **c** NEXAFS spectrum at the C-K edge of nc-Ni composites (0.8 vol.% C). Ni-C chemical bond, which corresponds to the absorption peak at 290.3 eV, is identified. **d** Representative tensile engineering stress-strain curves for nc-Ni and nc-Ni composites. Simultaneous enhancement of yield strength, fracture strength, and uniform elongation are observed for nc-Ni composites compared to pure nc-Ni. **e** Thermal stability of nc-Ni composites: cumulative area fraction of grain size of the as-fabricated nc-Ni composite (0.8 vol.% C) and after being annealed at various temperatures (973 K to 1193 K) for 1 h. No apparent grain growth was observed for nc-Ni composite after annealing at 1073 K for 1 h. The inset shows a representative bright-field TEM image for this annealing condition. **f** Electrical resistivity of nc-Ni and nc-Ni composite measured at the temperature range of 2 to 300 K. The electrical conductivity of nc-Ni composite (0.8 vol.% C) is ~77% higher than that of nc-Ni (3.9 S/m vs. 2.2 S/m) at room temperature (298 K).

The strategy of intragranular nanodispersion can be extended to other metallic systems. For example, we synthesized nc-Ni composites with the same approach (Methods). As illustrated in Fig. 5, The resultant nc-Ni composite (average grain size 56 ± 8 nm), exhibits the σ_y_ and σ_u_ of 2.2 ± 0.2 GPa and 2.8 ± 0.3 GPa, respectively (Fig. 5d), far above its pure nanocrystalline counterparts and even higher than those reported in Ni-based metallic glass^23^. Our nc-Ni composites also retain considerable tensile ductility (>5%). We further tested the thermal stability of nc composites via annealing and ion channelling imaging. For nc-Cu composites (0.8 vol.% C), no obvious grain coarsening was observed at the annealing temperature up to 0.72*T*_m_ for 1 h (where *T*_m_ is the melting temperature) (Fig. 3f). This upper limit of annealing temperature is considerably higher than the grain-coarsening temperature of the reference nc-Cu (about 0.3*T*_m_) and also surpasses the reported highest temperature that nc-Cu can sustain without coarsening (0.45*T*_m_)^38^. Moreover, our nc-Cu composite was measured to have an electrical conductivity of 41.8 ± 1.7 × 10^6^ S m^−1^ at room temperature, which is about 40% higher than that of pure nc-Cu with similar grain sizes (27.8 ± 1.2 × 10^6^ S m^−1^)^21^ (Methods). The high thermal stability and improved electrical conductivity were also observed in nc-Ni composites (0.8 vol.% C), which exhibit a stable nanograined structure up to 0.62*T*_m_ and 1.77-fold increase in electrical conductivity at room temperature (Fig. 5e, f).

To investigate the underlying mechanisms responsible for the high thermal stability, we conducted HAADF-STEM and APT characterization of the annealed nc-Cu composites (0.8 vol.% C) (Supplementary Fig. 11). Owing to highly stabilized unc, pinning of GBs through their interactions with numerous unc particles can be identified, thereby leading to negligible coarsening of nanograins at 973 K for 1 h. This result suggests that unc is likely to increase the energy barrier for GB migration and sliding. No marked impurity segregation was observed at GBs by APT analysis. Thus, the improved grain stability is not due to the solute drag effect of GBs. This is further supported by the control experiments of nc-Cu with a similar impurity level (Supplementary Table 1), which show substantially lower thermal stability (~0.3*T*_*m*_). Therefore, the high thermal stability of nc-Cu composites should be closely associated with the extremely dense unc distribution and the strong interfacial bonding of Cu-C^39,40^.

To summarize, our work represents an important step forward to overcome several major shortcomings of nanograined metals via a nanodispersion-in-nanograins strategy. We show that the uniform intergranular dispersion of carbon nanoparticles in nanocrystalline metals is an effective approach to realize enhanced work hardening and thus high ductility, in conjunction with improved thermal stability and electrical conductivity. These combined properties exceed those achieved by other nanostructuring strategies, including GB engineering, hierarchical microstructuring^41^, and nanotwinning^17,26^. Hence, nanodispersion in nanograins represents a promising approach that may be widely applicable to make ultra-strong, ductile, and stable metal nanocomposites for future structural and functional applications.

## Methods

### Material fabrication

#### Nanocrystalline Cu (nc-Cu) and nc-Cu composites

Graphene oxide (GO) can be absorbed on Cu flake surfaces via electrostatic interactions, as detailed in our previous work^15^. The as-fabricated GO-Cu composite flakes were annealed at 673 K for 1 h in a tube furnace under flowing H_2_/Ar (20 vol.% H_2_, flow rate: 200 sccm) to reduce GO, remove surface surfactant and minimize copper oxide that may have been produced during previous fabrication processes. The obtained reduced GO (RGO)-Cu composite powders were ball-milled in a Fritsch Pulverisette 6 high-energy planetary mono-mill with Fe-Cr stainless steel ball-to-powder weight ratio of 10:1. Before milling, a batch of pure Cu powder was added into the hardened chromium steel grinding bowl and pre-milled for 2 h, in order to clean the grinding media and reduce contaminations. The whole handling and milling processes were proceeded under the protection of high-purity argon (99.999+%). The milling time was 8 h at a speed of 300 rpm. Note that the purpose of ball-milling here is to refine the grain size of Cu powder while breaking down the graphene sheets to carbon nanoparticles. Welded powder pieces with flat geometry (diameter of ~2 mm and thickness between 200–500 μm) were obtained after the milling process, consistent with previous reports^21,42^. To avoid excessive temperature increase during milling, the grinding bowl was fan-cooled, and 30 min interval was used during ball-milling. The temperature of the milling bowl was monitored intermittently with an infrared thermometer (Lasergrip 800, Etekcity) and the maximum temperature that can be reached during milling was measured to be 328 K. To minimize oxidation, the as-milled pure Cu and composites were kept in a glove box with oxygen and water content less than 0.5 ppm. When the volume fraction of unc particles exceeds 0.8%, RGO tends to stack on metal nanoflakes, inhibiting the effective fragmentation and embedding of unc particles during ball milling. Before microstructure characterizations and mechanical testing, both pure Cu and composite samples were annealed at 423 K for 10 mins in an oven that sat inside a glove box. The purpose of this short annealing is to relieve the residual stresses caused by ball milling based on our XRD studies.

#### Nc-Ni and nc-Ni composites

The fabrication processes for nc-Ni and nc-Ni composites (0.8 vol.% C) were almost identical with Cu samples as described above, except that the average size of Ni powder was 3 μm (purity 99.9+%, Alfa Aesar). Nc-Ni and nc-Ni composites powders were annealed at 473 K for 10 mins in order to relieve residual stress. Mechanical and microstructural characterization procedures of Ni samples were identical with Cu samples.

### Composition analysis

The compositions of nc-Cu composites with different ultra-nano-carbon (unc) contents (0.4 vol.% and 0.8 vol.% C) and pure nc-Cu were analysed by inductively coupled plasma mass spectroscopy (ICP-MS, ThermoFisher i CAPQ) for metallic elements and instrumental gas analysis (IGA, Evans Analytical Group, LLC.) for light elements (hydrogen, carbon, nitrogen), after the dust and moisture removal. The total impurity content in the as-fabricated nc-Cu composites was found to be less than 0.1%.

### Carbon nanoparticle density measurements

To identify the density of carbon nanoparticle (C nanoparticle) in our nc-metal composites, we etched 20 grams of the nc-Cu composites (0.8 vol.% C) with ferric chloride solution (0.5 mol L^−1^). The matrix was entirely etched after a deposition time of 12 h, and C nanoparticles were agglomerated and deposited at the bottom of the solution. A thin film of agglomerated C nanoparticles with a diameter of 20 mm can then be obtained by vacuum filtration of the solution and rinsing with deionized water for five times. The film was carefully peeled off from the filter paper and dried at 333 K for 12 h in an oven that sat inside a glove box. The mass and thickness of the carbon paper can be measured by high-accuracy balance and SEM, respectively. We have estimated the density of C nanoparticle to be 2.24 ± 0.13 g cm^−3^ with three independent measurements. The measured density value was used to convert carbon content from the weight percent to the volume percent in the composites.

### Fabrication of bulk nanocrystalline samples

The ball-milled pure metal (Cu) and composite powders (nc-Cu composites) were densified to form bulk samples of pure metal and composite, respectively. Powders were first cold compacted into billets with diameter 40 mm via a custom-made compression machine that was placed inside a glove box under a pressure of 200 MPa. The billets were vacuum-packed before taken out from the glove box in order to avoid oxidation during the transfer. Vacuum hot pressing at 573 K and 600 MPa for 1 h was carried out to obtain pure metal and composite in bulk form with high density (>99%) without obvious grain growth. Dog-bone specimens with 8 mm gauge length, 2 mm width, and 1 mm thickness were cut from the bulk nanocrystalline samples by electro-discharge machining (EDM). Surfaces and sides of the tensile samples were polished with 360−4000 European grit metallographic silicon carbide papers, followed by polishing in 3 μm diamond suspensions. The final thickness of the tensile specimens was ~0.9 mm. Quasi-static uniaxial tensile tests were conducted in a universal testing machine (Instron 3344) at a constant strain rate of 5 × 10^−4^ s^−1^ at ambient temperature. Five tensile tests were carried out to obtain statistical values. Tensile elongations were measured using a static axial clip-on extensometer (Model 2630-101, Instron) with the displacement resolution of 1 μm.

### Measurement of thermal stability

Nc-metal (Ni and Cu) and nc-metal composites (Ni and Cu, 0.8 vol.% C) were annealed at different temperatures (573 K, 673 K, 773 K, 873 K, 973 K, 1073 K) for 1 h in order to evaluate their thermal stability. The annealing was performed in an argon environment. Significant grain growth occurs for pure nc-Cu and nc-Ni samples at temperatures of 573 K or above. We employed ion-channelling cross-sectional image to statistically calculate the grain size distribution of nc-Cu that processed at 673 K, the result of which was displayed in Fig. 3f. No apparent grain growth was observed for nc-Cu and nc-Ni composites annealed at temperatures below 973 K and 1073 K, respectively.

### Measurement of electrical conductivity

For electrical conductivities measurements, nc-Ni and nc-Ni composites (0.8 vol.% C) were measured by Physical Property Measurement System (PPMS) (PPMS-9T, Quantum Design) in the temperature range of 2–300 K, using a standard van der Pauw resistivity measurement method^43^. The sample dimension is 8 mm × 8 mm × 1 mm (length × width × height). Due to the low electrical resistance of pure Cu and nc-Cu composite, the PPMS measurements yielded rather scattered data. The electrical conductivities of nc-Cu and nc-Cu composites were therefore obtained by eddy current measurements (SMP 10, Fischer) with an accuracy of ±0.5%. Ten positions were tested for each sample (sample dimension: 30 mm × 30 mm × 2 mm) in order to obtain the statistically meaningful average value. The electrical conductivity was expressed in terms of International Annealed Copper Standard (IACS). High-purity annealed copper was used for calibration purposes. All samples used for electrical conductivity measurements were polished down to a metallurgical grit of 4000 SiC paper.

### Structural characterization

#### Transmission electron microscopy (TEM) and strain mapping

Bright-field TEM images of as-fabricated pure nc-Cu and nc-Cu composites were performed by JEOL 2100 F at the voltage of 200 keV. The scanning TEM (STEM) images were recorded in a Cs-corrected JEOL JEM-ARM 200 F at the voltage of 200 keV fitted with an imaging aberration corrector (CEOS). High-resolution high-angle annular dark-field STEM (HAADF-STEM) images, with a maximum point-to-point spatial resolution of 0.08 nm, were obtained with a convergence angle of 26 mrad and collection semi-angles from 50 to 180 mrad. TEM samples were prepared by the lift-out method using focused ion beam (FIB, Scios, FEI) and further nano-milling (Nanomill 1040, Fischione,) operated at 800 V was employed to remove the surface amorphous layer during FIB milling. The statistical calculation of size and location of unc in the composites were achieved by analysing at least 20 HADDF-STEM images. Strain mapping analysis was completed using geometrical phase analysis (GPA) software installed in Digital Micrograph (Gatan). The image drifts were corrected using a StackReg (Rigid Body) plugin in ImageJ software. The pinning probability of dislocations by unc particles was calculated by the number of particles that pinned dislocations divided by the total number of particles examined by TEM, in a particle size bin (1–1.5 nm, 1.5–2 nm, 2–2.5 nm, etc.).

#### SXRD and NEXAFS

Synchrotron x-ray diffraction (SXRD) pattern of nc-Cu composites (0.8 vol.% C) was collected at the Advanced Photon Source (APS) of Argonne National Laboratory at the beamline 11-ID-B, equipped with an amorphous silicon-based area detector (Perkin-Elmer). The x-ray beam energy was 100 keV (λ = 0.1238 Å) and the beam slot size was 0.5 × 0.5 mm^2^. The two-dimensional pattern was integrated in full ranges with Fit 2D to obtain the 1D diffraction pattern that is shown in Fig. 1e. The SXRD spectrum of nc-Ni composites (0.8 vol.% C) was obtained at the Shanghai Synchrotron Radiation Facility (SSRF) at the beamline BL14B1, equipped with a line detector (Mythen 1 K). The collection was preceded using a θ−2θ scanning mode, with the step size of 0.002^°^. The x-ray beam energy was 18 keV and the beam slot size was 1 × 1 mm^2^. C 1 s near-edge x-ray absorption fine-structure (NEXAFS) spectra of nc-Cu and nc-Ni composites were obtained at the National Synchrotron Radiation Laboratory (NSRL), China. The data were collected by a total electron yield (TEY) mode at the BL11U beamline, with a rectangular beam size of 0.2 × 0.1 mm^2^.

#### Raman spectroscopy

We used Raman spectroscopy to characterize the defect states of graphene in the composite powders (0.8 vol.% C). Both composite powders before and after the ball-milling were probed. Micro-Raman spectroscopy (Senterra R200-L, Bruker) with an excitation wavelength of 633 nm (1.96 eV) was used. A laser beam power of 10 mW and an exposition time of 10 s was employed so as to collect sufficient Raman intensity, as the embedding of unc into metal matrix drastically reduces the carbon signal. Five measurements were taken in each composite powder (i.e., before and after ball-milling) from different areas. The Raman peaks (D, G, and D^’^) were fitted with Lorentzian functions with the commercial software Origin 8, and we denote their intensities (height) as I(D), I(G), and I(D’). The I(D)/I(G) ratio was used to estimate the domain size of graphene, following the theory and equation described by our previous work^44^. For the graphene we used, the crystalline domain size was found to decrease from 1.3 nm to 1.1 nm after ball milling. Since our unc has an average size of ~2.5 nm, this suggests that a large fraction of unc is amorphous.

#### Atom probe tomography (APT)

The APT analyses were carried out in a Cameca LEAP 4000X SI under ultrahigh vacuum of ~2.5 × 10^−11^ torr. The specimen temperature is 40 K, and the target evaporation rate of 0.5% per pulse, under UV laser pulsing with an energy of 40 pJ. APT specimens were prepared with a FIB system, where a very low accelerating voltage (5 kV) and beam current (5 pA) were employed in the final milling step to minimize Ga^+^ implantation. The CAMECA integrated visualization and analysis software IVAS 3.6.12 was used for data processing and three-dimensional atomic reconstruction. For the APT analysis, note that there is an overlap between the field-evaporated ions ^27^Al^+^ and ^54^Fe^2+^ at 27 Da on mass spectrum. We carefully examined the isotopic ratio of Fe ions at 27, 28 and 29 Da to evaluate how many Al ions were evaporated at 27 Da in the form of ^27^Al^+^, and found that the concentration is marginal (or non-detectable). Two additional peaks at 9 Da (Al^3+^) ions and 13.5 Da (Al^2+^) ions were observed in our APT result, confirming the existence of Al trace in the sample. Supplementary Movie 1 shows APT three-dimensional reconstruction from the analysis of nc-Cu composites (0.8 vol.% C), demonstrating the uniform embedding of the nanoparticles. The threshold for the iso-composition surface is 0.25 at.% C.

We estimated the average C nanoparticle spacing in our nc-Cu composites (0.8 vol.% C) based on the APT results. The volume of the APT tip, as displayed in Fig. 2e, f, was calculated to be 1.59 × 10^5^ nm^3^. As the number of unc contained in the tip was 124, the average volume (*V*) shared by each unc was 1282 nm^3^. Therefore, we obtained an average particle spacing *l* (*l* = *V*^ 1/3^) of 10.9 nm. This value agrees reasonably well with that estimated according to the volume fraction. The unc particles, with the statistically calculated average size (*D*) of 2.5 nm from APT results, possess 0.8 vol.%. The average particle spacing can thus be estimated as *l*  = $\left(\frac{\pi {D}^{3}}{6\times 0.008}\right)$^1/3^ = 10.7 nm. We used the average particle spacing of 10.7 nm for our MD simulation.

### Mechanical testing

The micro-tensile specimens with square cross section (1.3 μm × 1.3 μm) and a gauge length of ~ 4 μm were fabricated by the FIB system. The geometry of gauge section was designed in such a way (see Supplementary Fig. 3) that an early fracture of specimen near the ear section can be avoided. A rectangular trench, with dimension of 60 μm × 120 μm × 10 μm (length × width × depth), was milled from the specimen surface, then one micro-tensile sample was fabricated in the middle of another trench (90 μm × 90 μm × 10 μm) that situated above the previously milled one. This sample milling strategy ensures a good view of specimen during the in situ mechanical testing in SEM (Scios, FEI). A low voltage of 5 keV and low current of 5 pA were employed to mill specimens to the final dimension in order to alleviate the Ga^+^ implantation (Supplementary Fig. 4d, e). The Ga ion trajectories and distributions using current mill conditions were simulated by SRIM software. Uniaxial tensile tests were carried out on an in situ micro-/nano-mechanical tester (Nanoflip, Nanomechanics®) at room temperature, inside an SEM chamber. The tensile grips were milled from a conical conductive diamond tip. Sample holder was titled by 8 degrees before mechanical testing in order to obtain clear view of both tensile sample and grips. Tensile tests were conducted at a nominal strain rate of 5 × 10^−4^ s^−1^. Five tests were performed to obtain the statistics and the engineering stress-strain responses were displayed in Supplementary Fig. 4a–c. The load-displacement data and the real-time morphology of tensile samples were recorded synchronously. InView software (Nanomechanics Inc.) was employed to monitor and analyse the mechanical testing data. Micro-tensile samples with a gauge cross-sectional area 2 × 2 μm^2^ were also milled and tested at a strain rate of 5 × 10^−4^ s^−1^, to investigate the sample size effect on tensile properties.

#### Tensile strain measurements

We corrected the stress-strain curves by applying the elastic modulus of composites, which was measured their hot-pressed samples with a nanoindenter (G200, Agilent) equipped with a Berkovich indentation tip. The accuracy of our approach was verified by directly comparing the obtained strain value with that derived from the change in gauge length in recorded video measurements in SEM. Supplementary Movie 2 shows one example. The tensile elongation to failure for nc-Cu composite (0.8 vol.% C) was estimated to be 0.141 in the video, which correlates well with the Young’s modulus corrected one (0.138). The comparison tensile testing of pure nc-Cu is shown in Supplementary Movie 3.

### Computational modelling

#### Molecular dynamic (MD) simulations

Molecular dynamics simulations were performed using LAMMPS^45^ to study the interaction between dislocations and unc particles. The atomic interactions in the Cu-C system were modelled by combining the embedded atom method (EAM) potential^46^ for Cu-Cu interaction, the Tersoff potential^47^ for C-C interaction, and the Lennard-Jones potential^48^ for Cu-C interaction. A slab of face-centered cubic Cu single crystal was constructed with the dimension of 46.3 nm × 63.2 nm × 10.7 nm. The corresponding crystal orientation was X-[110], Y-[001], and Z-$[1\bar{1}0]$. Periodic boundary conditions were imposed in both the X and Z directions, while the Y surface of the slab was free to relax. A spherical C particle with a diameter *d* of 2.5 nm was embedded in the simulation cell, representing a periodic array of C particles with a spacing of 10.7 nm along the Z direction due to the periodic boundary condition imposed. This setup represents an equivalent C concentration of 0.8% as studied in our experiment. Both the crystalline C particle with a diamond cubic lattice structure and the amorphous C particle with a disordered atomic structure were modelled. Different C particle sizes and the effect of misfit strains between the C particle and Cu matrix were studied. Either one or two 60° dislocations of the 1/2〈110〉{111} type were embedded in the simulation cell; the dislocation line was aligned along the Z-direction of $[1\bar{1}0]$ and located on a {111} slip plane intersecting the C particle. To control the position and character of a dislocation embedded in the simulation cell, we first imposed the atomic displacements of this dislocation according to its elastic solution and then relaxed the simulation cell to obtain a dissociated full dislocation consisting of a pair of leading and trailing partials with a stacking fault in between. The simulation cell was subjected to an imposed tensile strain up to 0.1% along the X-direction, which was sufficient to drive the dislocation(s) to bypass the periodic array of C particles. We obtained similar results by both molecular statics simulations (i.e., a sequence of energy minimization by the conjugate gradient method) and molecular dynamics simulations at low temperatures. The von Mises equivalent atomic strain field in Fig. 4g was produced by OVITO^49^. In addition, we studied the effect of interface strength between the Cu matrix and C particle on the critical shear stress for sequential bypassing of C particles by five dislocations. By adjusting the parameters in the Lennard-Jones potential for Cu-C interaction, we studied the effective interface strength of 0.008 and 0.114 J/m^2^, respectively, as shown in Supplementary Fig. 7.

#### First principles calculations

First principles calculations were performed using Cambridge serial total energy package (CASTEP) based on density functional theory (DFT) for spin-polarized electronic structures and total energies^50,51^. The calculations for exchange and correlation were based on generalized gradient approximation (GGA)^52^ in the Perdew-Burke-Ernzerhof (PBE) form^53^. The energy cutoff for the plane-wave basis about the ultrasoft pseudopotentials was set at 440eV^54,55^, and all integrations over the Brillouin zone were done using the Monkhorst–Pack scheme with one to four k-points in the relevant irreducible wedge^56^. After relaxation, the quantum-mechanical force on each atom was below 0.02eVÅ^−1^.

#### A two-stage hardening model

We developed a two-stage hardening model as described in the main text. In this model, a classical rate-dependent plastic formulation is used. Under uniaxial tension, the strain rate $\dot{\varepsilon }$ is decomposed as $\dot{\varepsilon }={\dot{\varepsilon }}^{e}+{\dot{\varepsilon }}^{\,p}$, where ${\dot{\varepsilon }}^{e}$ is the elastic strain rate and ${\dot{\varepsilon }}^{\,p}$ is the plastic strain rate. The stress rate $\dot{\sigma }$ is given ${\dot{\sigma}}=E {\dot{\varepsilon }}^{e}$, where *E* is Young’s modulus. The tensile stress $\sigma$ and the resolved shear stress $\tau$ on the primary slip system are related by $\sigma=M\tau$, where *M* is the Taylor factor. Correspondingly, the tensile plastic strain rate ${\dot{\varepsilon }}^{\,p}$ and the shear plastic strain rate ${\dot{\gamma }}^{p}$ are related by ${\dot{\gamma }}^{\,p}=M{\dot{\varepsilon }}^{\,p}$, so that $\sigma {\dot{\varepsilon }}^{\,p}=\tau {\dot{\gamma }}^{\,p}$. As discussed in the main text, the density of geometrically necessary dislocations from dispersed unc particles is

1 $${\rho }^{{{{{{\rm{G}}}}}}}=f{\gamma }^{p}/(bd)$$

where *f* is the volume fraction of unc particles, *b* is the Burgers vector length, and *d* is the unc particle size. The plastic shear rate ${\dot{\gamma }}^{p}$ is determined by the resolved shear stress.

2 $${\dot{\gamma }}^{p}={\dot{\gamma }}_{0}^{p}{\left(\frac{\tau }{{\tau }_{{{{{{\rm{T}}}}}}}}\right)}^{\frac{1}{m}}$$

where ${\tau }_{{{{{{\rm{T}}}}}}}$ is the total slip resistance on the primary slip system, *m* is the rate sensitivity, and ${\dot{\gamma }}_{0}^{p}$ is the reference shear plastic strain rate. We used the following general relation to represent the total slip resistance for both stage I and II hardening

3 $${\tau }_{{{{{{\rm{T}}}}}}}={\tau }_{{{{{{\rm{y}}}}}}}+{\tau }_{{{{{{\rm{I}}}}}}}^{{{{{{\rm{UNC}}}}}}}+{\tau }_{{{{{{\rm{II}}}}}}}^{{{{{{\rm{UNC}}}}}}}$$

where ${\tau }_{{{{{{\rm{y}}}}}}}$ is the shear resistance at the yield point for pure nc-Cu, ${\tau }_{{{{{{\rm{I}}}}}}}^{{{{{{\rm{UNC}}}}}}}$ is the shear resistance of stage I hardening arising from unc particles and becomes saturated at the end of stage I hardening; and ${\tau }_{{{{{{\rm{II}}}}}}}^{{{{{{\rm{UNC}}}}}}}$ is the additional shear resistance from stage II hardening. The rate of rapidly growing ${\tau }_{{{{{{\rm{I}}}}}}}^{{{{{{\rm{UNC}}}}}}}$ due to GNDs arising from unc particles is expressed as

4 $${\dot{\tau }}_{{{{{{\rm{I}}}}}}}^{{{{{{\rm{UNC}}}}}}}=k({\tau }_{{{{{{\rm{I}}}}}},{{{{{\rm{sat}}}}}}}^{{{{{{\rm{UNC}}}}}}}-{\tau }_{{{{{{\rm{I}}}}}}}^{{{{{{\rm{UNC}}}}}}}){\dot{\gamma }}^{p}$$

where *k* is the constant characterizing the rate of stage I hardening; ${\tau }_{{{{{{\rm{I}}}}}},{{{{{\rm{sat}}}}}}}^{{{{{{\rm{UNC}}}}}}}$ is the saturated value of ${\tau }_{{{{{{\rm{I}}}}}}}^{{{{{{\rm{UNC}}}}}}}$ at the end of stage I hardening (taken approximately as 4% plastic strain), and is given by ${\tau }_{{{{{{\rm{I}}}}}},{{{{{\rm{sat}}}}}}}^{{{{{{\rm{UNC}}}}}}}={\beta }_{1}\mu b{\rho }_{4\%}^{{{{{{\rm{G}}}}}}}D$, where ${\beta }_{1}$ is the constant coefficient, ${\rho }_{4\%}^{{{{{{\rm{G}}}}}}}$ is the density of GNDs arising from unc particles at the plastic strain 4%, and *D* is the grain size. The additional shear resistance from stage II hardening ${\tau }_{{{{{{\rm{II}}}}}}}^{{{{{{\rm{UNC}}}}}}}$ is estimated as ${\tau }_{{{{{{\rm{II}}}}}}}^{{{{{{\rm{UNC}}}}}}}={\beta }_{2}\mu b\sqrt{{\rho }^{{{{{{\rm{G}}}}}}}}$, where ${\beta }_{2}$ is the constant coefficient. To simulate the stress-strain responses of nc-Cu composites and pure nc-Cu, we used the finite difference method to integrate the above rate relations as a function of time. The applied strain rate $\dot{\varepsilon }$ was taken as $1\times {10}^{-3}\,{{{{{{\rm{s}}}}}}}^{-1}$ and the integration time increment is 0.001 s. The parameters used in this model are listed in Supplementary Table 2. Figure 4l shows the stress-strain curves given from this model for nc-Cu composites and pure nc-Cu, which capture the major trend in experiments.

### Intragranular dispersion mechanisms of carbon nanoparticles

Pre-dispersion of RGO on metal surfaces is vital to achieve unc dispersion inside grains. We examined the microstructure of nc-Cu composites prepared by conventional mechanical alloying, and found that in samples after 48 h-ball milling, unc particles were not incorporated into grain interiors. Hence, the pre-dispersion strategy plays a critical role in the effective dispersion of unc particles. Moreover, fragmentation of RGO could also be facilitated by the relatively low fracture strength of RGO with defects (e.g., oxygen-containing groups and vacancies)^57,58^. To further show the advantage of unc dispersion with RGO as C precursor, we chose different types of nanocarbons (e.g., 1-D C nanotube and 2-D C nanosheets) with less defects as C precursor and investigated their dispersion in nc-Cu. Single-walled C nanotubes (SWCNTs) or few-layer graphene nanosheets (FLGNSs) with high crystallinity were pre-dispersed on Cu nanoflakes. Then the composite powders were ball-milling-processed using the same milling conditions as described previously. APT and HAADF-STEM results revealed that these nanocarbons were mainly distributed along grain boundaries, rather than embedded inside grains, as shown in Supplementary Fig. 2. The poor dispersion of nanocarbons could be attributed to insufficient defects in SWCNTs and FLGNSs, which reduce the density of metal-C chemical bonds, increase the interfacial energy, and thereby lower the effectiveness of nanocarbon embedding.

### Comparison with tensile data in the literature

In Fig. 3d, we compared the tensile properties of our nc-Cu composites with a number of representative Cu systems in the literature, including pure Cu and Cu alloys, as well as high-performance Cu containing nanotwins or other types of heterogonous structures. References for the data points in Fig. 3d are listed as follows: conventional pure Cu [■^59^, ▲^60^, ●^61^, ▼^62^, ◄^63^], heterogeneous Cu [♦^64^, ^1^, ^65^, □^66^, △^67^, ◯^68^], nanotwinned Cu [▽^26^], nanostructured Cu alloys [◁^69^, ◊^70^, ^71^], complexion-engineered nanograined Cu alloys [^71^], and nc-Cu obtained in this study (★).

## Supplementary information

Supplementary Information

Description of Additional Supplementary Files

Supplementary Movie 1

Supplementary Movie 2

Supplementary Movie 3

## Data Availability

All data supporting the findings of this study are included in this article (and its Supplementary information file).
