# Supplementary material for: A nanodispersion-in-nanograins strategy for ultra-strong, ductile and stable metal nanocomposites
Source: Nat Commun. 2022 Sep 23;13:5581. doi: 10.1038/s41467-022-33261-5 (PMC9508098; doi:10.1038/s41467-022-33261-5)
Supplement: Supplementary file 1 — Supplementary Information [file 41467_2022_33261_MOESM1_ESM.pdf]

Supplementary Information

A nanodispersion-in-nanograins strategy for ultra-strong, ductile and stable metal nanocomposites

Zan Li[1#], Yin Zhang[2#], Zhibo Zhang[3,4], Yi-Tao Cui[5], Qiang Guo[1], Pan Liu[1], Shenbao Jin[6], Gang Sha[6], Kunqing Ding[2], Zhiqiang Li[1], Tongxiang Fan[1], Herbert M. Urbassek[4], Qian Yu[7], Ting Zhu[2*], Di Zhang[1*] & Y. Morris Wang[8*]

[1]State Key Laboratory of Metal Matrix Composites, Shanghai Jiao Tong University, Shanghai 200240, China.

[2]Woodruff School of Mechanical Engineering, Georgia Institute of Technology, Atlanta, Georgia 30332, USA.

[3]Institute of Materials and Processing, Guangdong Academic of Science, Guangzhou 510000, China.

[4]Physics Department and Research Center OPTIMAS, University Kaiserslautern, Erwin-Schrödinger-Straße, D-67663 Kaiserslautern, Germany.

[5]Synchrotron Radiation Laboratory, Laser and Synchrotron Research Centre (LASOR), The Institute for Solid State Physics, The University of Tokyo, Hyogo 679-5165, Japan.

[6]School of Materials Science and Engineering, Gleiter Institute of Nanoscience, Nanjing University of Science and Technology, Nanjing 210094, China.

[7]Center of Electron Microscopy and State Key Laboratory of Silicon Materials, School of Materials Science and Engineering, Zhejiang University, Hangzhou 310027, China.

[8]Department of Materials Science and Engineering, University of California, Los Angeles, CA 900095, USA.

[#]These authors contribute equally. *Correspondence to: ting.zhu@me.gatech.edu (TZ); zhangdi@sjtu.edu.cn (DZ); ymwang@ucla.edu (YMW)

Content:

Supplementary Figures 1 - 11

Supplementary Tables 1 - 2

Supplementary Movies 1 - 3

Supplementary Figures

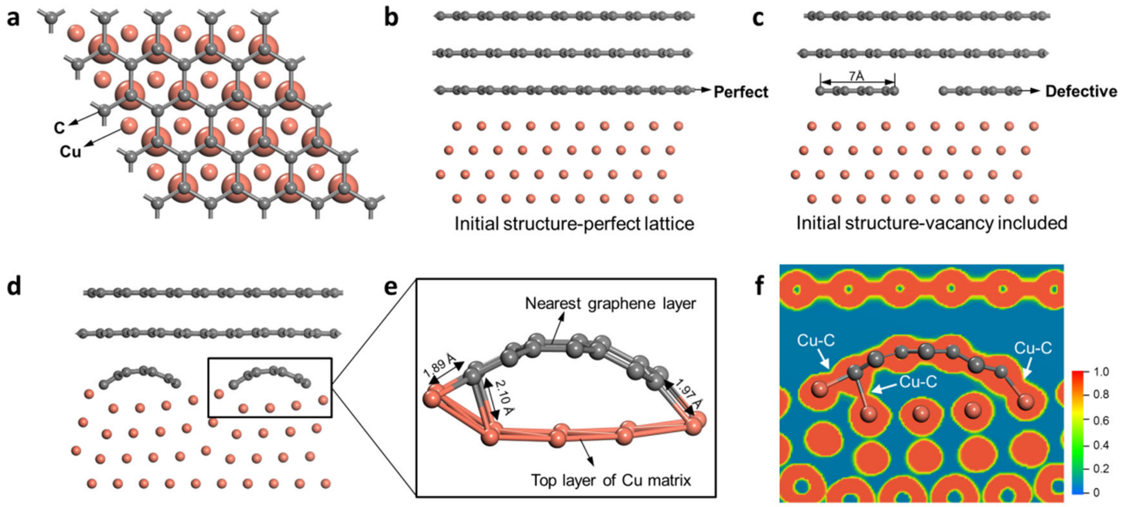

Supplementary Figure 1 | Density functional theory (DFT) calculation of metal-C bond formation. a, Top view of a model system of Cu-graphene interface. **b,** Side view of the initial structure of the interface between Cu and perfect graphene. **c**, Same as **b** except for defective graphene, where C atoms are removed at the interface. **d,** DFT result after relaxation showing the bending of a defective graphene plane due to strong Cu-C interactions at defective sites. **e,** Enlarged view of the boxed region in **d**, showing the largely shorten atomic distance between Cu and defective carbon atoms. **f,** Corresponding electron localization function (ELF) map showing electron localization between C and Cu atoms and thus suggesting the formation of Cu-C chemical bonds.

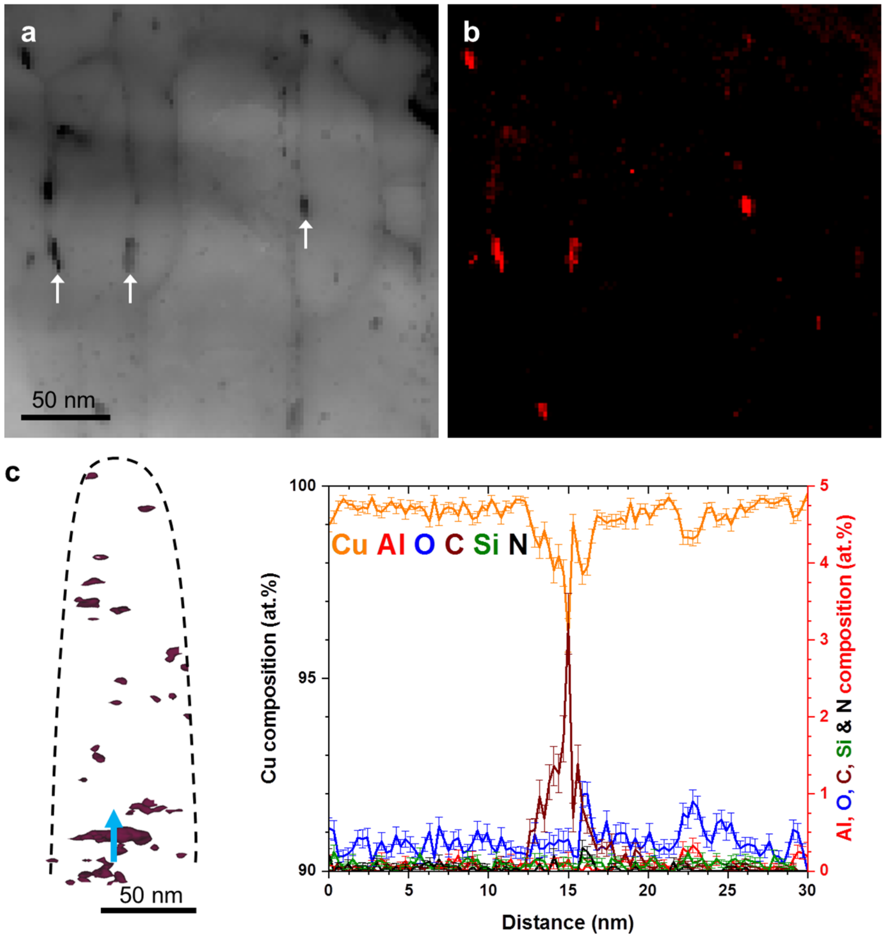

Supplementary Figure 2 | Microstructure characterization of nc-Cu composites reinforced with high-crystalline carbon precursor. a, HAADF-STEM image, **b,** carbon spectrum imaging map, and **c,** APT three-dimensional reconstruction map of nc-Cu composite with SWCNTs (0.8 vol.%) as carbon precursor. This composite was obtained using the same fabrication processes as nc-Cu composites. As indicated by white arrows in **a**, carbon particles were found to mainly distribute at grain boundaries (GBs) in this composite. The proximity histogram across the matrix and a carbon particle shown in **c** (the right diagram) reveals the C-rich nature of the particle. The threshold for the iso-composition surface in APT analysis is 0.25 at.% C. The APT experiment was carried out at the Max-Planck-Institut für Eisenforschung in a Cameca LEAP 5000XR under an ultrahigh vacuum of approximately 2.5×10^{-11} torr.

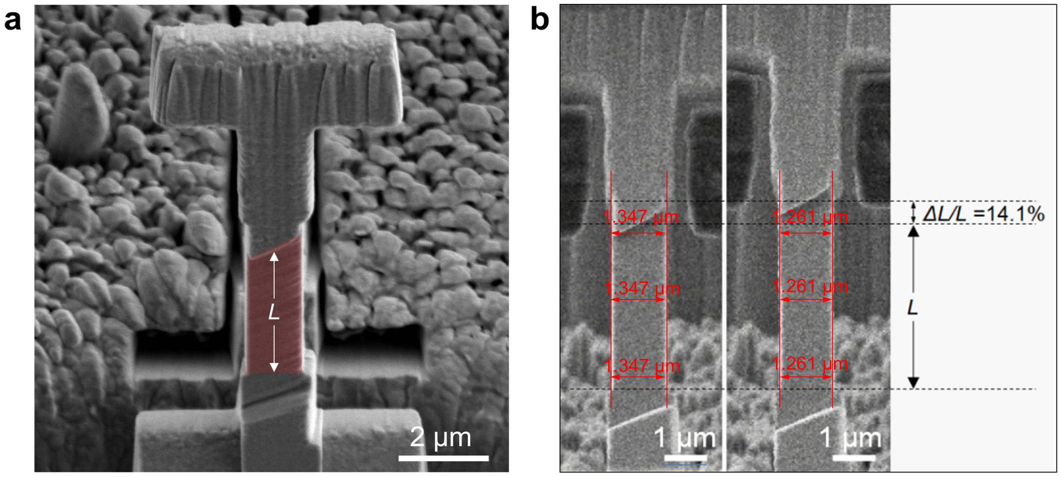

Supplementary Figure 3 | *In situ* SEM tensile testing of nc-Cu composites. a, Front-view SEM image showing the overall geometry of an as-fabricated tensile specimen (gauge width, ~1.3 μm). Note that the image was taken at a 52° tilt angle. The region experienced large tensile deformation is indicated by brown colour. The gauge length (*L*) is indicated by two white arrows and used for the estimate of tensile strain. **b,** *In situ* SEM images of tensile testing of a nc-Cu composite (0.8 vol.% C), showing the specimen before test (left) and before fracture (right). The sample was tested at a strain rate of 5×10^{-4} s^{-1} and the tensile-elongation-to-failure ($\Delta L/L$) was estimated to be 14.1%. The entire process of tensile testing can be viewed in Supplementary Movie S2.

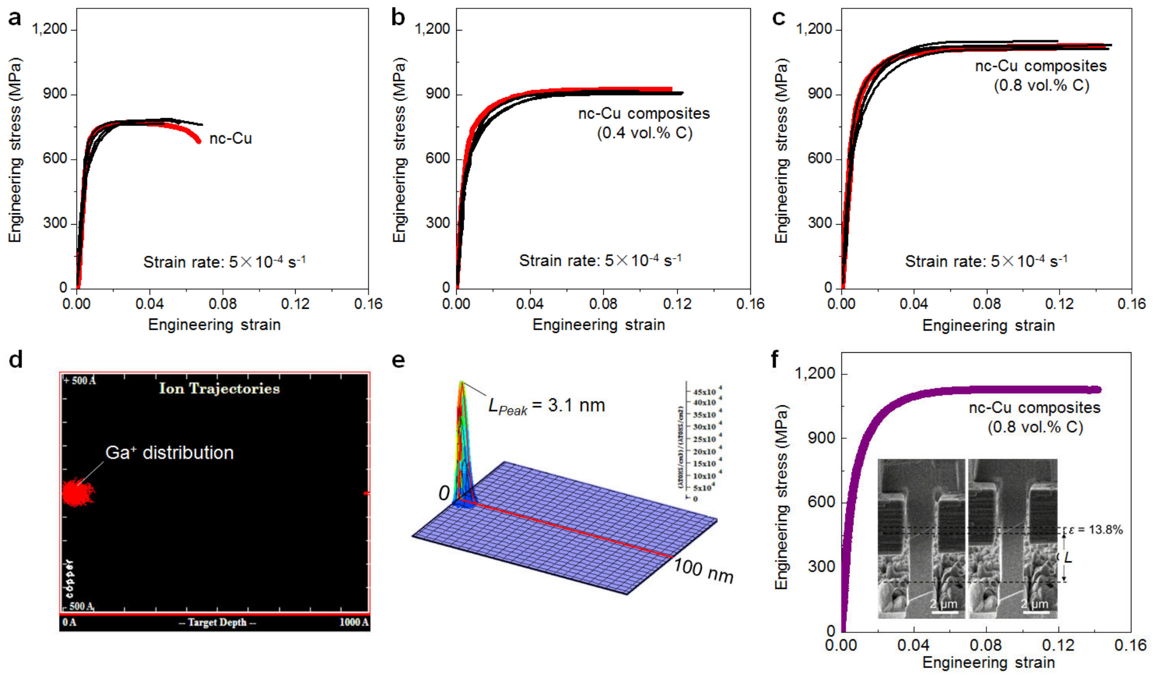

Supplementary Figure 4 | Reproducibility of tensile results. a-c, Engineering stress-strain curves of five tests for pure nc-Cu (**a**), nc-Cu composites (0.4 vol.% C) (**b**), and nc-Cu composites (0.8 vol.% C) (**c**). Good reproducibility is observed for all three types of materials. The representative tensile curves shown in Fig. 3a were highlighted in red. **d,** Simulation results of Ga^+ trajectories in Cu at an accelerating voltage of 5 keV, revealing the injected Ga^+ are distributed mainly within 10 nm below Cu surface. The number of the incident Ga^+ is 5000. **e,** Ga^+ implantation profile in Cu at the 5 keV accelerating voltage. The peak of the penetration depth was found to be 3.1 nm, which is negligibly small compared to the sample thickness. **f,** Representative engineering stress-strain curve of nc-Cu composites (0.8 vol.% C) with larger sizes (gauge width, ~2.0 μm), showing similar mechanical responses to those in **c**. Inset shows the morphology of the larger-sized sample before the test (left) and before the fracture (right). Sample was tested at a strain rate of 5×10^{-4} s^{-1}.

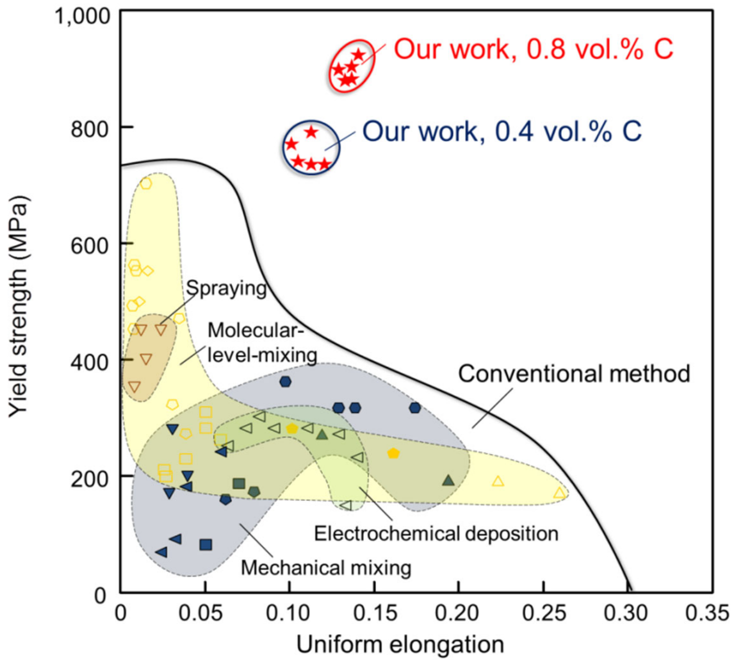

Supplementary Figure 5 | Comparison of tensile properties with literature data. Yield strength versus uniform elongation of our nc-Cu composites, compared to those of graphene-reinforced Cu composites obtained with various fabrication technologies. Sources of data are as follows: mechanical mixing (■[1], ▲[2], ⬣[3], ⬟[4], ▼[5], ◀[6]), molecular-level-mixing (⬟[7], □[8], △[9], ⬡[10], ⬠[11], ◇[12]), spraying (▽[13]) and electrochemical deposition (◁[14]).

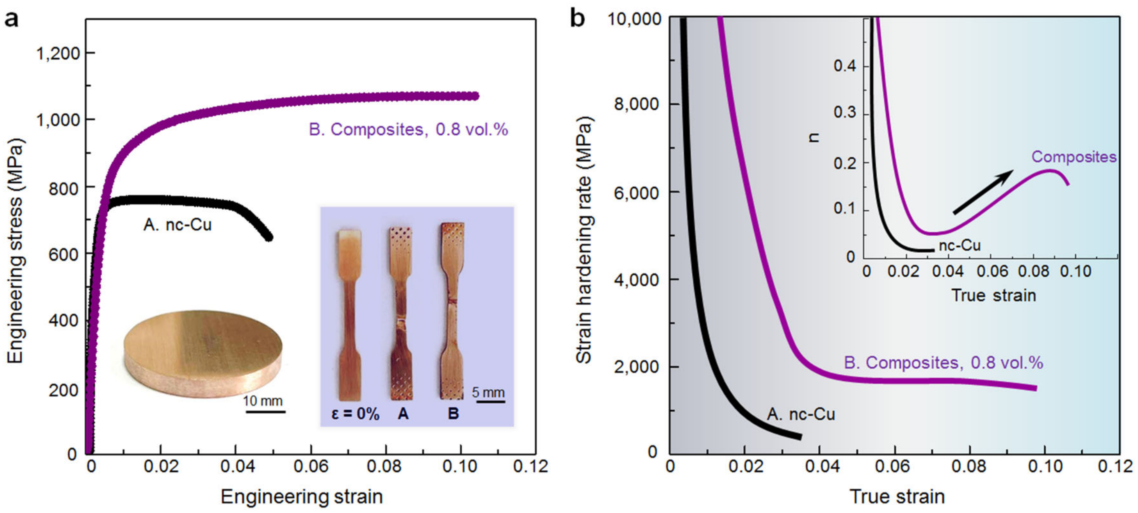

Supplementary Figure 6 | Mechanical properties of bulk pure nc-Cu and nc-Cu composite. a, Tensile engineering stress-strain curves for bulk pure nc-Cu (A) and nc-Cu composite (0.8 vol.% C) (B). All tensile tests were performed at a strain rate of 5×10^{-4} s^{-1} and room temperature. The left inset shows a micrograph of the consolidated bulk sample (diameter: 40 mm), while the right inset shows the micrographs of tensile fractured pure nc-Cu (middle) and unc-dispersed nc-Cu composite (right) specimens, together with a tensile specimen before straining (left). **b,** Experimentally measured strain hardening rate $d\sigma/d\varepsilon$ (with σ and ε being the true stress and true strain, respectively) for nc-Cu composites and pure nc-Cu. Inset shows the strain hardening exponent ($n = d(\ln\sigma)/d(\ln\varepsilon)$) as a function of strain, where an 'up-turn' behaviour is visible for bulk nc-Cu composites.

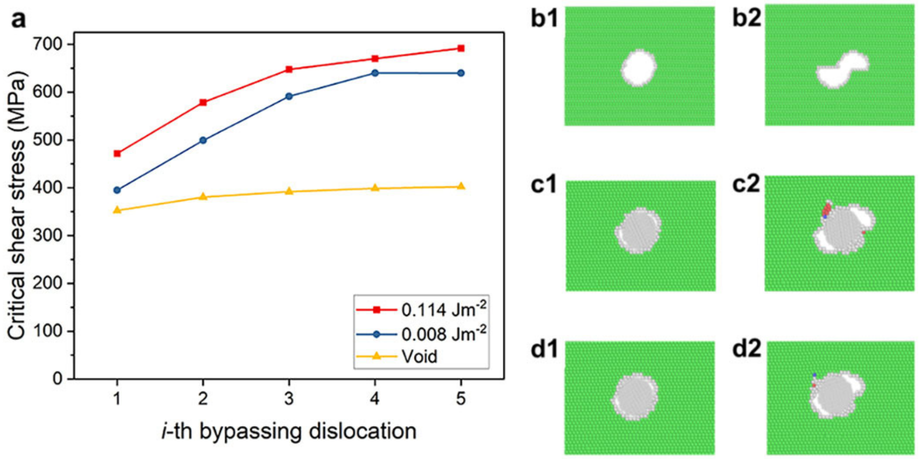

Supplementary Figure 7 | Atomistic simulation results showing the effect of interfacial strength on unc strengthening behaviour. a, Critical shear stress as five dislocations sequentially bypass around unc particles. Comparison is made for dislocation bypassing a void, a unc particle with a low interface strength of 0.008 Jm^{-2}, and a unc particle with a high interface strength of 0.114 Jm^{-2}, respectively. The size of the void and unc particle is 2.5 nm. **b1, b2**, Snapshot after the 1st and 5th dislocation bypasses a void, producing a small and a large step on the void surface, respectively. **c1, c2**, Snapshot after the 1st and 5th dislocation bypasses a unc particle with the low interface strength, producing a small and a large step at the particle-matrix interface, respectively. **d1, d2**, Same as **c1**, **c2**, respectively, except for producing relatively small steps at the particle-matrix interface, due to the increased interface strength compared to the case of **c1** and **c2**.

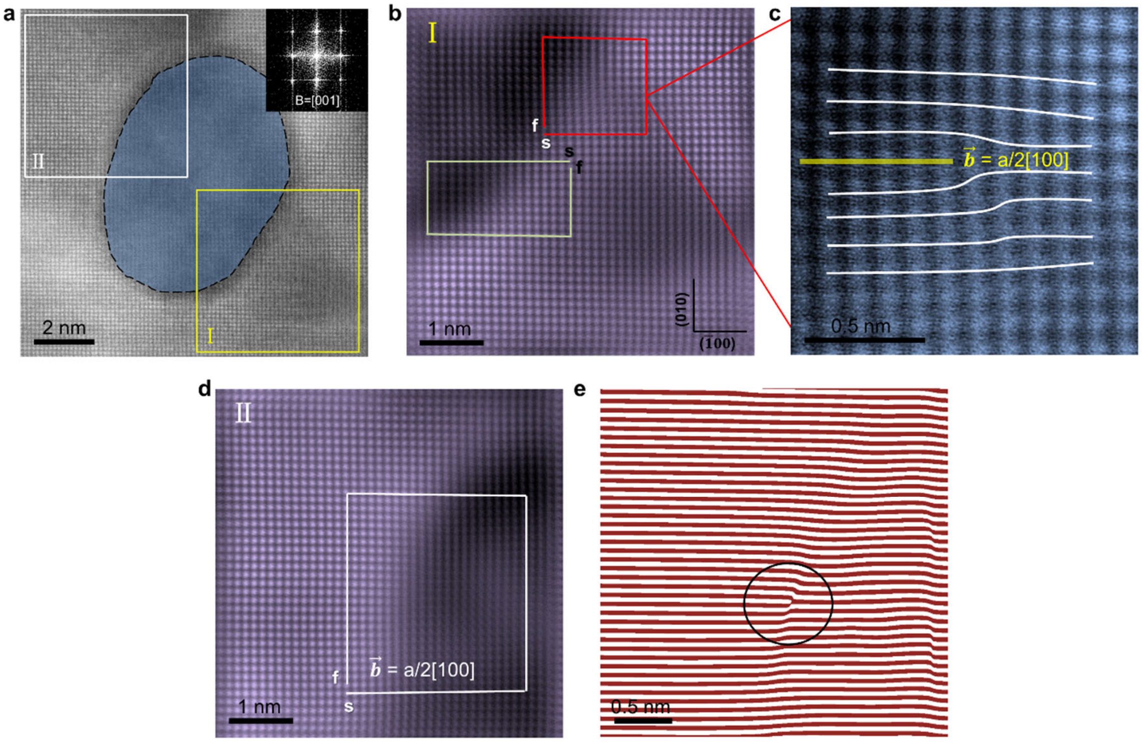

Supplementary Figure 8 | Dislocation blockage by unc particles in a deformed nc-Cu composite. a, A high-resolution HAADF-STEM image of the local atomic structure near a unc particle taken along the [001] zone axis, as confirmed by the fast Fourier transformation (FFT) pattern in the inset. The particle is highlighted in blue, with its boundary indicated by the black dashed line. Two regions (I and II) are chosen for microstructural analysis. **b,** A high magnification HAADF-STEM image of region I**.** Two dislocations with the in-plane Burgers vector of a/2[100] can be identified near the nanoparticle by the red and yellow Burgers circuits, respectively. **c,** An enlarged view of the dislocation core structure, showing an additional row of atoms corresponding to an extra half plane. **d,** A high magnification HAADF-STEM image of region II. The white Burgers circuit shows a dislocation with the in-plane Burgers vector of a/2[100] near the nanoparticle. **e,** The corresponding inverse Fourier-filtered lattice structure of region II, where an additional row of atoms (as indicated by a black circle) is identified. These dislocations likely correspond to the projection of 3D full dislocations with the Burgers vector of a/2<110>{111}.

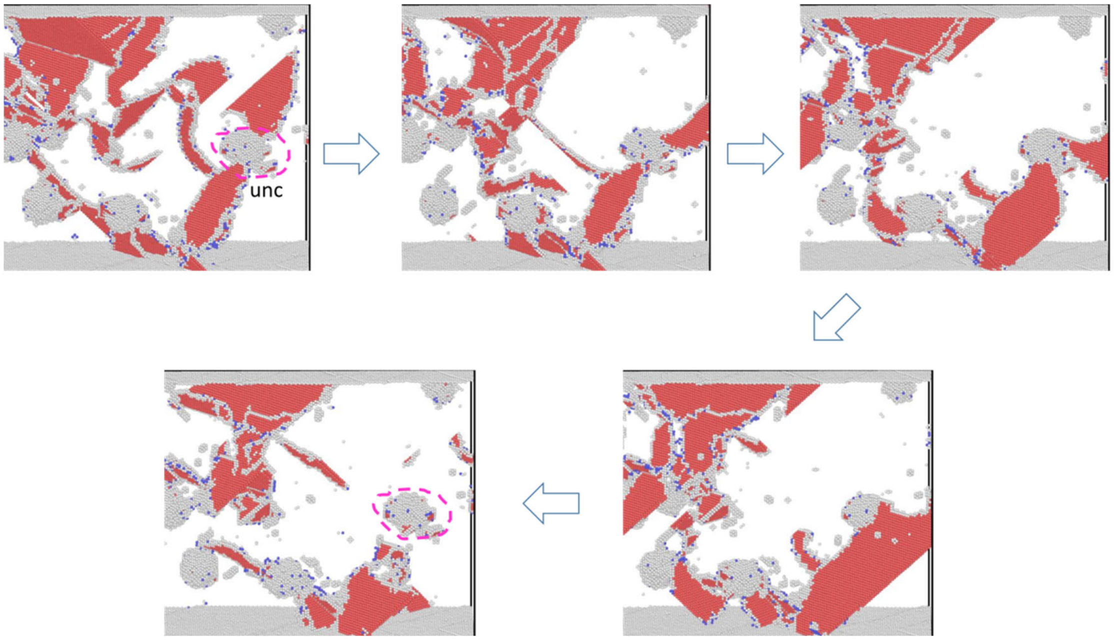

Supplementary Figure 9 | MD simulation results of dislocation-obstacle interaction in Cu containing randomly distributed unc particles. These MD snapshots highlight the dynamic processes of dislocation pinning by a unc particle (indicated by a dashed-line oval) and subsequent dislocation breakaway from the particle. From these MD results, we identified the representative unit process of dislocation pinning and bypassing, which have been further studied in a more controllable manner in Fig. 4h and i.

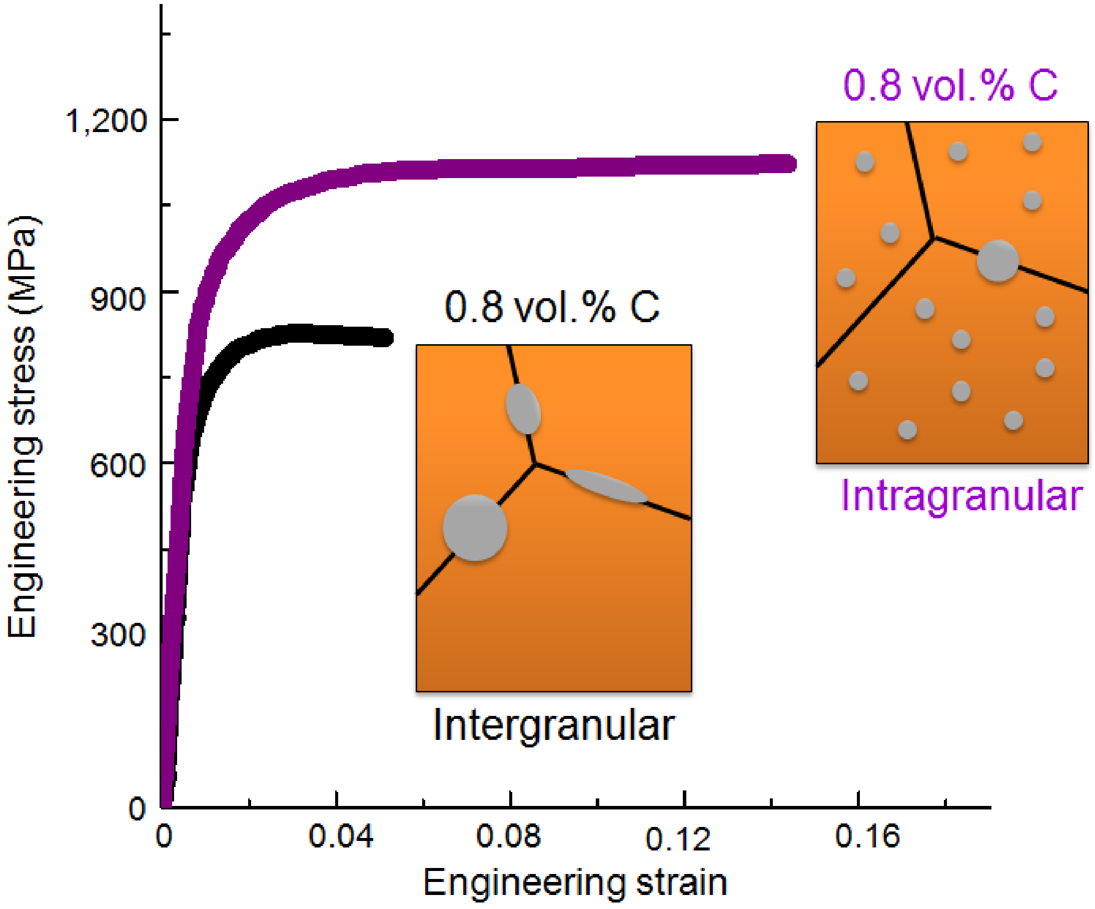

Supplementary Figure 10 | Tensile engineering stress-strain curves of nc-Cu composites reinforced with intragranular and intergranular C nanoparticles (0.8 vol.%), respectively. The distribution of C nanoparticles is closely associated with the bonding nature of Cu-C, as discussed in the Methods section. For the intragranular dispersion of C nanoparticles, the strengthening and work hardening capacities are greatly improved due to strong dislocation-particle interactions.

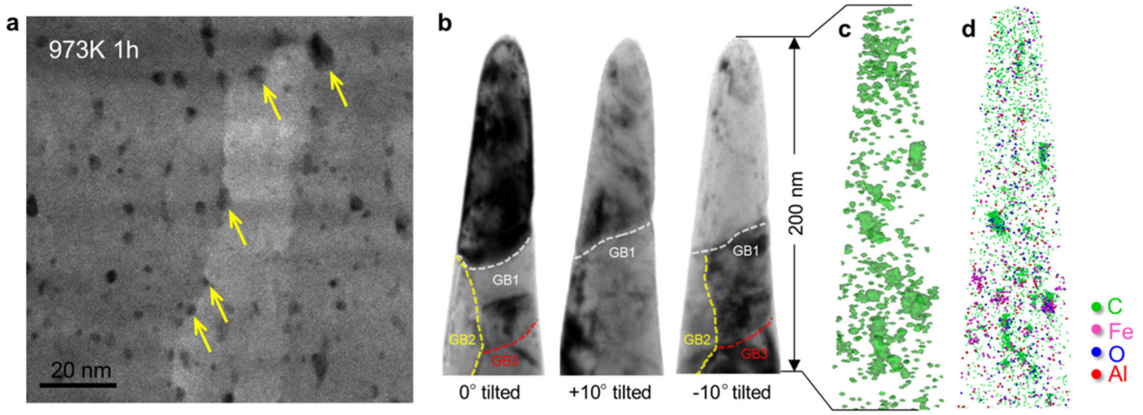

Supplementary Figure 11 | Thermal stability of nc-Cu composites. a, HAADF-STEM image of nc-Cu composites (0.8 vol.% of C) after thermal annealing at 973K for 1h. The GB was pinned by unc particles as indicated by black arrows. **b,** BF-TEM image of the APT tip tilted by different angles (0°, +10° and -10°), where three GBs can be identified. **c,** 3D reconstructed C atom map with 0.25 at.% C iso-composition surface. The average particle size is 2.6 ± 1.2 nm, showing negligible particle coarsening after annealing. **d,** 3D reconstructed atom map showing C and other detectable impurities (Al, Fe and O). Despite the presence of spatial correlation between Fe and C, atomic segregation at GBs is insignificant. Note that the contents of impurities are limited (25 ppm, 190 ppm and 19 ppm for Al, Fe and O, respectively)

Supplementary Tables

Supplementary Table 1 | Compositions of nc-Cu and nc-Cu composites. Impurity concentrations are shown, with their mean standard deviations obtained from two independent measurements.

Element	nc-Cu (ppm)	Composites (0.4 vol.% C) (ppm)	Composites (0.8 vol.% C) (ppm)
H	15±2	12±2	17±3
C	20±3	960±16	2,065±20
N	62±5	52±5	72±4
Al	22±9	26±5	26±5
Ca	29±4	31±5	38±5
Co	4±1	4±1	14±2
Cr	48±5	57±4	96±5
Fe	166±12	164±5	174±9
K	21±4	22±4	21±3
Mg	13±1	24±2	18±2
Mn	19±3	14±1	35±2
Mo	7±2	8±1	9±1
Na	16±0	9±2	14±2
Ni	25±3	35±2	30±3
Ti	11±2	22±1	15±1
Zn	17±2	15±1	18±0
**Cu**	**Bal.**	**Bal.**	**Bal.**

Note that the oxygen impurity was not analysed since it is sensitive to sample preparation for IGA measurements.

Supplementary Table 2 | Parameters used in the two-stage hardening model.

E	μ	b	M	$\dot{\gamma}_0^p$
117 GPa	44.7 GPa	0.256 nm	2.7	1×10^{-3} s^{-1}
m	k	β_1	β_2	σ_y
0.06	74 MPa	0.6	0.35	780 MPa
D	$\rho_{4\%}^{\mathrm{G}}$ (0.4% unc)	$\tau_{\mathrm{I,sat}}^{\mathrm{UNC}}$ (0.4% unc)	$\rho_{4\%}^{\mathrm{G}}$ (0.8% unc)	$\tau_{\mathrm{I,sat}}^{\mathrm{UNC}}$ (0.8% unc)
60 nm	2.5×10^{14} m^{-2}	38.1 MPa	5.0×10^{14} m^{-2}	76.3 MPa

Supplementary references

1. Li, M., Che, H., Liu, X., Liang, S. & Xie, H. Highly enhanced mechanical properties in Cu matrix composites reinforced with graphene decorated metallic nanoparticles. *J. Mater. Sci.* **49,** 3725-3731 (2014).
2. Tang, Y., Yang, X., Wang, R. & Li, M. Enhancement of the mechanical properties of graphene–copper composites with graphene–nickel hybrids. *Mater. Sci. Eng. A* **599,** 247-254 (2014).
3. Kim, W. J., Lee, T. J. & Han, S. H. Multi-layer graphene/copper composites: Preparation using high-ratio differential speed rolling, microstructure and mechanical properties. *Carbon* **69,** 55-65 (2014).
4. Jiang, R., Zhou, X., Fang, Q. & Liu, Z. Copper–graphene bulk composites with homogeneous graphene dispersion and enhanced mechanical properties. *Mater. Sci. Eng. A* **654,** 124-130 (2016).
5. Yang, M., Weng, L., Zhu, H., Fan, T. & Zhang, D. Simultaneously enhancing the strength, ductility and conductivity of copper matrix composites with graphene nanoribbons. *Carbon* **118,** 250-260 (2017).
6. Chu, K., Wang, F., Wang, X. & Huang, D. Anisotropic mechanical properties of graphene/copper composites with aligned graphene. *Mater. Sci. Eng. A* **713,** 269-277 (2018).
7. Hwang, J. *et al.* Enhanced mechanical properties of graphene/copper nanocomposites using a molecular-level mixing process. *Adv. Mater.* **25**, 6724-6729 (2013).
8. Chen, F. *et al.* Effects of graphene content on the microstructure and properties of copper matrix composites. *Carbon* **96,** 836-842 (2016).
9. Zhang, D. & Zhan, Z. Strengthening effect of graphene derivatives in copper matrix composites. *J. Alloy. Compd.* **654,** 226-233 (2016).
10. Yang, Z. *et al.* Preparation mechanism of hierarchical layered structure of graphene/copper composite with ultrahigh tensile strength. *Carbon* **127,** 329-339 (2018).
11. Si, X. *et al.* Effect of carbide interlayers on the microstructure and properties of graphene-nanoplatelet-reinforced copper matrix composites. *Mater. Sci. Eng. A* **708,** 311-318 (2017).
12. Wang, L. *et al.* Graphene-copper composite with micro-layered grains and ultrahigh strength. *Sci. Rep.* **7,** 41896 (2017).
13. Liu, X., Wei, D., Zhuang, L., Cai, C. & Zhao, Y. Fabrication of high-strength

graphene nanosheets/Cu composites by accumulative roll bonding. *Mater. Sci. Eng. A* **642,** 1-6 (2015).

14. Song, G. *et al.* Direct determination of graphene amount in electrochemical deposited Cu-based composite foil and its enhanced mechanical property. *RSC Adv.* **7,** 1735-1742 (2017).
