# Supplementary material for: A nanodispersion-in-nanograins strategy for ultra-strong, ductile and stable metal nanocomposites
Source: Nat Commun. 2022 Sep 23;13:5581. doi: 10.1038/s41467-022-33261-5 (PMC9508098; doi:10.1038/s41467-022-33261-5)
Supplement: Supplementary file 2 — Description of Additional Supplementary Files [file 41467_2022_33261_MOESM2_ESM.pdf]

Description of Additional Supplementary Files

File Name: Supplementary Movie 1
Description: APT three-dimensional reconstruction from analysis of nc-Cu composite (0.8 vol.% C). The threshold for the iso-composition surface is 0.25 at.% C.

File Name: Supplementary Movie 2
Description: *In situ* SEM tensile test of nc-Cu composite (0.8 vol.% C). The video is accelerated by ten times.

File Name: Supplementary Movie 3
Description: *In situ* SEM tensile test of pure nc-Cu. The video is accelerated by ten times.
